# A Dense Genetic Linkage Map for Common Carp and Its Integration with a BAC-Based Physical Map

**DOI:** 10.1371/journal.pone.0063928

**Published:** 2013-05-21

**Authors:** Lan Zhao, Yan Zhang, Peifeng Ji, Xiaofeng Zhang, Zixia Zhao, Guangyuan Hou, Linhe Huo, Guiming Liu, Chao Li, Peng Xu, Xiaowen Sun

**Affiliations:** 1 Centre for Applied Aquatic Genomics, Chinese Academy of Fishery Sciences, Beijing, China; 2 Heilongjiang Fisheries Research Institute, Chinese Academy of Fishery Sciences, Harbin, China; 3 College of Life Science and Technology, Dalian Ocean University, Dalian, China; 4 Chinese Academy of Sciences Key Laboratory of Genome Sciences and Information, Beijing Institute of Genomics, Chinese Academy of Sciences, Beijing, China; Temasek Life Sciences Laboratory, Singapore

## Abstract

**Background:**

Common carp (*Cyprinus carpio*) is one of the most important aquaculture species with an annual global production of 3.4 million metric tons. It is also an important ornamental species as well as an important model species for aquaculture research. To improve the economically important traits of this fish, a number of genomic resources and genetic tools have been developed, including several genetic maps and a bacterial artificial chromosome (BAC)-based physical map. However, integrated genetic and physical maps are not available to study quantitative trait loci (QTL) and assist with fine mapping, positional cloning and whole genome sequencing and assembly. The objective of this study was to integrate the currently available BAC-based physical and genetic maps.

**Results:**

The genetic map was updated with 592 novel markers, including 312 BAC-anchored microsatellites and 130 SNP markers, and contained 1,209 genetic markers on 50 linkage groups, spanning 3,565.9 cM in the common carp genome. An integrated genetic and physical map of the common carp genome was then constructed, which was composed of 463 physical map contigs and 88 single BACs. Combined lengths of the contigs and single BACs covered a physical length of 498.75 Mb, or around 30% of the common carp genome. Comparative analysis between common carp and zebrafish genomes was performed based on the integrated map, providing more insights into the common carp specific whole genome duplication and segmental rearrangements in the genome.

**Conclusion:**

We integrated a BAC-based physical map to a genetic linkage map of common carp by anchoring BAC-associated genetic markers. The density of the genetic linkage map was significantly increased. The integrated map provides a tool for both genetic and genomic studies of common carp, which will help us to understand the genomic architecture of common carp and facilitate fine mapping and positional cloning of economically important traits for genetic improvement and modification.

## Introduction

Common carp (*Cyprinus carpio*) originated in Eurasia and became one of the most important cultured fish species in the world with an annual global production of 3.4 million metric tons that accounts for nearly 14% of all freshwater aquaculture production in the world [1]. In addition to its economical importance, common carp is also considered as a model species for various studies on ecology [2], environmental toxicology [3]–[4], developmental biology [5], immunology [6], evolutionary genomics [7], nutrition [8] and physiology [3]. With increasing demand for the genome resources of this species efforts had been made in the past decades to unveil and understand the genome of common carp. As a result, the available genomic resources for common carp research have increased and include a large number of polymorphic loci, genetic markers [6], [9]–[13], databases [14], genetic linkage maps for multiple generations [15]–[17], expressed sequence tags (ESTs) and transcriptome sequences [18], [19], a bacterial artificial chromosome (BAC) library [20], BAC end sequences (BES) [21], BAC-based physical maps [22], cDNA microarrays [23]–[25] and whole genome exome data [26]. These resources have been used to analyze important genes and quantitative trait loci (QTL) related to various economic traits [27]–[29] and for comparative analysis with other cyprinids [30].

The first generation of BAC-based physical maps of common carp was constructed using High Information Content Fingerprints (HICF) technology [31], which generated a total of 67,493 BAC clones assembled into 3,696 contigs with an average length of 476 kb and a N50 length of 688 kb representing approximately 1.76 Gb of the carp genome [22]. In parallel, the genetic linkage map of common carp was constructed based on 617 microsatellite markers [32]. However, it is important to integrate two types of maps and facilitate genome studies ranging from chromosome-scale genome sequence assembly to position-based gene identification to improve important traits.

Integration of both linkage and physical maps, is considered as an important step toward whole genome sequencing and assembly, especially for species with large and complex genomes, although it is a challenge to achieve complete genome-scale integration. Both physical and genetic linkage maps have been constructed for many aquaculture species in the past decades [33]–[39] and these maps have been partially integrated in catfish, rainbow trout and Atlantic salmon. For example, the first generation of integration map of rainbow trout was composed of 238 BAC contigs anchored to the genetic map, covering over 10% of the rainbow trout genome [40]. BAC-anchored SNP markers have been developed and used to anchor 73 BAC contigs to the Atlantic salmon genetic map [41]. In catfish, a total of 2,030 BAC end sequence (BES)-derived microsatellites from 1,481 physical map contigs were developed and used for map integration. These anchored 44.8% of the catfish BAC physical map contigs covering 52.8% of the genome [33], [42]–[46]. The genetic map is generally based on genome-wide markers, and the physical map is constructed based on the alignment of short DNA fragments. Integration of the two types of map will provide the essential tools to understand genomes in different scales, and will also facilitate whole genome sequencing and assembly. For instance, the integrated map of common carp in this study provides many more sequence tags for comparative mapping with the zebrafish genome, and gives us a more comprehensive understanding on genome evolution of common carp.

Here, we report the integration of physical and genetic maps of common carp based on BAC-anchored microsatellite and SNP markers. A large number of novel microsatellite markers were developed from BESs and mapped into linkage groups. In addition, BAC clones that harbor previously mapped markers were identified by homolog comparison between marker sequences and BESs. Comparative mapping between the genomes of common carp and zebrafish was then performed based on the integrated map of common carp.

## Results and Discussion

### Physical-map-contig-anchored Microsatellite Markers

In our previous study, a total of 10,355 BESs were identified to contain microsatellite motifs from 65,720 common carp BESs, of which 5,150 BESs had sufficient flanking sequences for PCR primer design [21]. A number of BAC-anchored microsatellite markers were developed for genetic linkage map construction, with some of them having been used for genetic linkage map construction previously [32], which provided us with the first batch of BAC-anchored genetic markers for map integration. However, these markers were not anchored to the physical map as they were developed before physical map construction. In the current study, we used these BAC-anchored microsatellite markers for map integration. A total of 244 BAC-anchored microsatellite markers were successfully mapped onto the genetic linkage map, anchoring 169 physical map contigs and 46 single BAC clones, equivalent to 144.06 Mb of the common carp genome.

In addition to these 244 markers, in order to map large physical map contigs to the genetic linkage map, we selected 629 BAC-anchored microsatellite loci from the 230 largest physical map contigs for marker development (Table S1). Of these contig-anchored microsatellites, 550 markers were genotyped in the mapping panel. A total of 226 showed the presence of polymorphisms, including 206 with a single amplified region and 10 with duplicated patterns that were scored as two markers. After linkage analysis, a total of 206 informative markers were assigned to the genetic linkage map of common carp, which updated the previous genetic linkage map and integrated 166 physical map contigs into the genetic linkage map to a total length of 258.28 Mb (Table 1).

**Table 1:** Summary of BAC-anchored microsatellite and SNP markers used for map integration.

Marker Source	Number of markers	Physical length (Mb) (Number of contigs or BACs)
Pre-developed BAC-anchored microsatellite markers	244	144.06 (169 contigs and 46 BACs)
Contig-anchored	198	137.55 (169 contigs)
Single BAC-anchored	46	6.50 (46 BACs)
Newly developed contig-anchored microsatellite markers	206	258.28 (166 contigs)
Number of microsatellite loci tested	629	
Monomorphic in mapping panel	324	
Amplification failed	79	
Duplicated	10	
Informative for linkage analysis	226	
Mapped to linkage groups	206	
SNP markers associated with BES	130	69.55
Contig-anchored	92	64.23 (92 contigs)
Single BAC-anchored	38	5.32 (38 BACs)
Other microsatellite markers associated with BES	40	26.86
Contig-anchored	36	26.30 (36 contigs)
Single BAC-anchored	4	0.56 (4 BACs)
Total	620	1498.75 (463 non-redundant contigs and 88 BACs)

1 Note: The sum-length of all integrated contigs and BACs after removing redundancy.

All information on BAC-anchored microsatellites is listed in the supporting information that includes marker ID, primer sequences, BAC ID, GenBank accession number, physical map contig ID and physical length (Table S2). A flowchart is included as a supplement to interpret all marker resources for the map integration (Figure S3).

### Single Nucleotide Polymorphism (SNP) Markers

Recently, over 5,000 SNP markers were developed randomly and genotyped in a subset (107 individuals) of a F1 mapping panel using restriction-site associated DNA (RAD) technology [47]. The short sequences of these SNP markers were aligned to the BES database and a total of 137 SNP markers were thus mapped to physical map contigs and single BAC clones. These SNP markers were not selected based on contig length and position, but were randomly sampled along the genome and mapped to physical map contigs and single BAC clones. Linkage analysis thus mapped these 130 SNP markers onto the genetic linkage map and integrated 92 physical map contigs and 38 single BAC clones, spanning a total of 69.55 Mb of the common carp genome (Table S3).

### Genetic Mapping and Updating the Genetic Linkage Map

**Figure 1:**
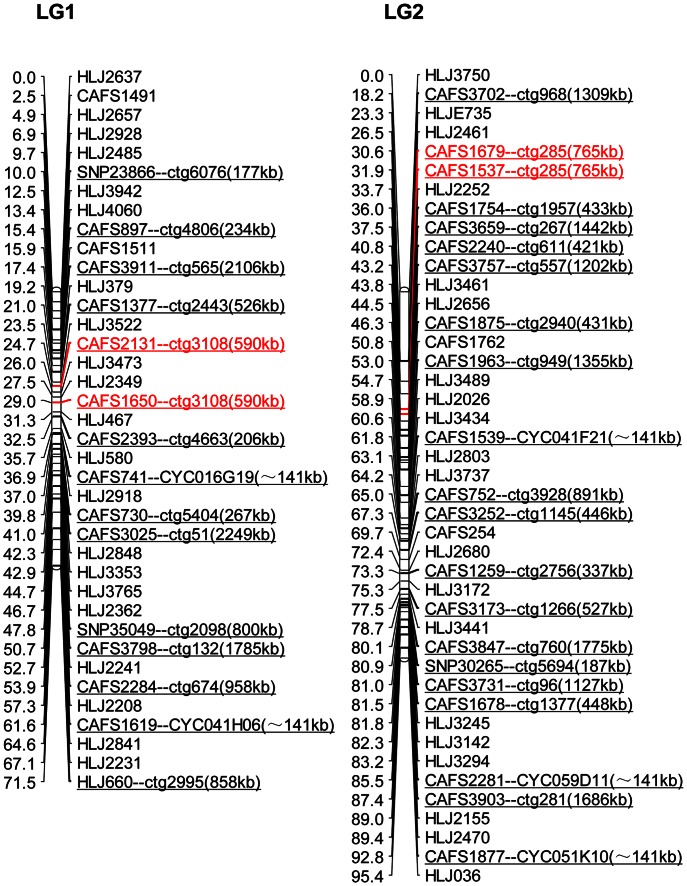
Linkage groups 1 and 2 of common carp are shown as an example. Annotation of the physical map contig or BAC clone linked to the marker are connected to the marker name (e.g. CAFS3025–ctg51 or CAFS1619–CYC041H06) and brackets around these markers give each contig/clone physical size. Annotation of “kb” means kilo base pairs and “∼141 kb” means approximately equal to 141 kilobase pairs. Red font markers and corresponding annotations represent those markers from the same linkage group linked to the same physical map contig. Distances between markers are shown in cM. All 50 linkage groups of common carp are presented in Figure S1.

A total of 1,359 polymorphic genetic markers, including previously mapped markers, newly developed markers and SNP markers, were used for linkage analysis and updating the genetic linkage map. There were 150 markers unmapped with a LOD score of 5.0 or excluded because of genetic segregation distortion. A total of 1,209 genetic markers, including 130 SNPs and 1,079 microsatellite markers, were finally mapped to 50 linkage groups (LGs). The updated genetic map had the highest marker density reported thus far, spanning 3,565.9 cM of the common carp genome. The genetic length of each linkage group ranged from 17.3 cM (LG50) to 126.7 cM (LG3) with an average length of 71.32 cM (Table 2). LG1 and LG2 are presented in Figure 1 as an example on which physical map contig IDs and BAC IDs were annotated to each anchored marker. All 50 linkage groups are presented in Figure S1. All genetic markers and their genotypes are listed in Table S4.

**Table 2:** Characteristics of the marker distribution among 50 linkage groups of common carp and their integrated physical length.

Linkage group	BES-anchored markers	Other markers	Total markers	Genetic Length (cM)	Average spacing (cM)	Maximum spacing (cM)	Anchored physical length (Mb)
LG1	15	23	38	71.527	1.933	4.469	11.038
LG2	21	22	43	95.398	2.271	18.168	15.205
LG3	19	20	39	126.716	3.335	21.306	13.768
LG4	18	16	34	91.198	2.764	8.132	14.691
LG5	17	15	32	77.483	2.499	18.28	12.727
LG6	13	19	32	84.551	2.727	16.205	9.385
LG7	19	16	35	53.407	1.571	5.657	18.451
LG8	11	14	25	70.365	2.932	14.304	10.843
LG9	10	13	23	91.522	4.16	17.264	8.665
LG10	21	13	34	80.977	2.454	13.346	23.482
LG11	15	13	28	67.203	2.489	8.153	11.077
LG12	13	14	27	73.801	2.839	8.345	12.11
LG13	24	19	43	70.092	1.669	15.483	16.959
LG14	11	11	22	55.163	2.627	8.683	7.856
LG15	7	14	21	104.901	5.245	34.385	5.739
LG16	11	17	28	73.158	2.71	19.206	8.617
LG17	19	10	29	74.529	2.662	7.163	15.376
LG18	8	11	19	108.209	6.012	24.542	7.934
LG19	16	11	27	88.88	3.418	12.229	10.545
LG20	13	11	24	62.509	2.718	22.738	13.301
LG21	9	11	20	83.293	4.384	12.485	7.811
LG22	12	19	31	101.361	3.379	14.472	11.58
LG23	11	15	26	60.024	2.401	10.906	8.727
LG24	13	9	22	71.157	3.388	10.147	9.968
LG25	10	8	18	69.281	4.075	10.713	7.915
LG26	10	10	20	70.518	3.711	12.747	8.047
LG27	13	13	26	61.281	2.451	13.058	10.957
LG28	13	7	20	72.796	3.831	23.9	7.972
LG29	12	14	26	71.95	2.878	25.105	15.723
LG30	21	16	37	85.511	2.375	13.187	18.624
LG31	9	23	32	81.673	2.635	21.287	6.624
LG32	15	7	22	75.25	3.583	14.948	13.75
LG33	9	9	18	59.809	3.518	10.018	7.09
LG34	8	10	18	76.005	4.471	18.808	7.461
LG35	14	6	20	67.051	3.529	14.84	12.033
LG36	10	8	18	64.688	3.805	17.252	17.27
LG37	8	7	15	31.54	2.253	5.68	7.28
LG38	13	7	20	55.411	2.916	10.263	11.98
LG39	9	11	20	33.866	1.782	11.244	5.749
LG40	5	7	12	77.752	7.068	29.19	3.626
LG41	4	4	8	73.751	10.536	29.536	2.03
LG42	23	9	32	64.868	2.093	15.102	21.75
LG43	12	7	19	54.718	3.04	14.509	15.686
LG44	7	13	20	75.497	3.974	12.068	9.05
LG45	12	12	24	105.152	4.572	30.679	8.915
LG46	6	5	11	28.436	2.844	8.323	5.006
LG47	9	5	14	61.469	4.728	12.059	6.783
LG48	3	4	7	39.198	6.533	10.09	3.618
LG49	12	10	22	53.701	2.557	10.147	10.725
LG50	7	1	8	17.315	2.474	9.799	9.233
Total	620	589	1209	3565.911			1540.752

1 Note: The sum-length of all linkage groups integrated contigs and BACs with redundancy.

### Physical Map Localization of Non-BAC-derived Microsatellite Markers

On the updated genetic map, 565 microsatellite markers previously developed from multiple resources (except the BAC resource) were used, including microsatellite enriched libraries and whole genome shotgun sequences, which were not anchored to BACs or physical map contigs. Sequences of these microsatellite markers were aligned to the BES database, which anchored 40 microsatellite markers to BAC clones or physical map contigs, thereby integrating 26.86 Mb of the common carp genome (Table 3).

**Table 3:** The BLAST results of HLJ microsatellite loci aligning with the BES database.

Marker ID	E-Value	BES ID	Contig ID	Physical length (kb)
HLJ1147	5.00E-93	CYC037J02.r	850	436
HLJ3563	2.00E-48	CYC085N09.f	252	837
HLJE170	3.00E-36	CYC054K09.r	538	1,235
HLJ3000	3.33E-13	CYC026H08.f	1227	1,419
HLJ3953	1.00E-73	CYC137I16.f	94	1,369
HLJ3852	7.00E-36	CYC075H13.f	2736	1,105
HLJ434	8.00E-140	CYC056C03.r	537	1,164
HLJ2910	2.00E-18	CYC046J22.f	1325	230
HLJ3601	4.00E-13	CYC055A09.f	152	527
HLJ2312	1.00E-23	CYC075F09.r	5317	428
HLJ456	3.00E-60	CYC032F08.r	386	708
HLJ3586	3.00E-78	CYC087E18.r	863	977
HLJ1098	3.00E-13	CYC029N04.f	1375	1,197
HLJ3444	1.00E-19	CYC090O05.r	1681	381
HLJ1266	1.50E-18	CYC134P01.r	2620	704
HLJ3515	5.00E-13	CYC033P02.f	7	910
HLJ2364	4.00E-95	CYC023I03.f	5195	146
HLJ3370	2.00E-51	CYC028A12.f	5117	280
HLJ495	1.50E-38	CYC092F11.r	1759	488
HLJE499	2.00E-76	CYC029C02.f	1748	217
HLJ3816	3.00E-39	CYC053P03.r	1474	731
HLJ1148	5.00E-12	CYC061H24.f	656	1,409
HLJ1163	2.00E-11	CYC100G13.f	806	1,015
HLJ1473	2.00E-11	CYC085H10.f	6397	196
HLJ2744	2.00E-23	CYC073M15.f	4552	346
HLJ3293	1.00E-49	CYC139A16.r	1205	605
HLJ3369	3.00E-41	CYC030N07.f	616	707
HLJ3526	1.00E-21	CYC100K09.f	167	955
HLJ3573	7.00E-14	CYC073H08.f	4917	583
HLJ3619	1.00E-33	CYC065K19.f	72	1,185
HLJ3675	2.00E-17	CYC038O24.f	7543	211
HLJ3948	3.00E-13	CYC061H24.f	656	1,409
HLJ3999	9.00E-17	CYC011A20.r	416	912
HLJ586	3.00E-27	CYC090A13.r	6842	157
HLJ660	9.00E-14	CYC073B03.f	2995	858
HLJE302	3.00E-29	CYC090I03.r	959	260
HLJ311	4.00E-13	CYC021F04.f	N/A	141
HLJ3995	2.00E-54	CYC039H14.r	N/A	141
HLJ3134	6.00E-64	CYC094N11.f	N/A	141
HLJ3937	4.00E-22	CYC074E22.f	N/A	141
Total				26860

### Genetic and Physical Map Integration

A total of 463 physical map contigs were integrated into the genetic linkage map by mapping 532 microsatellites and SNP markers to the linkage groups using multiple approaches, bringing 15,129 BAC clones and 19,506 BAC-end sequences onto the genetic linkage map. In addition, 88 single BACs with 175 BESs were anchored to the genetic linkage map by 88 markers. The combined lengths of 463 physical map contigs were 340,577 consensus bands (CB) or 486.34 Mb based on the conversion ratio of 1.428 kb per CB in the physical map of common carp [22]. Further, the 88 single BACs anchored onto the genetic linkage map also covered a physical length of 12.41 Mb when the average insert length of 141 kb for each BAC was counted. Thus the integration of physical and genetic linkage maps covered 498.75 Mb, which is about 30% of the common carp physical map and genome based on the estimated genome size using flow cytometry evidence (∼1.7 Gb; Table S5). Locations of the markers were annotated on the physical map and can be accessed from the physical map web-viewer (http://genomics.cafs.ac.cn/fpc/WebAGCoL/Carp/WebFPC/). The length of the anchored physical map contigs ranged from 129 kb to 3,122 kb with an average length of 1,050 kb. The number of anchored contigs per linkage group ranged from 3 to 19 with an average of 10, and the integrated physical length per linkage group ranged from 2.03 Mb to 23.48 Mb with an average length of 10.82 Mb (Table 2). There were 33 anchored physical map contigs containing more than one anchoring marker on the genetic linkage map, which means the orientation of these physical map contigs could be fixed on the genetic linkage map.

Linkage and physical map integration with BAC-anchored markers could also assess the quality of the physical map assembly and genetic linkage map construction. The markers on 13 anchored physical map contigs were mapped to two different linkage groups, implying the potential errors of either physical map assembly or genetic linkage mapping. We presumed that if the genetic linkage map was constructed correctly in this study, the estimated error rate of the physical map assembly would be 3% (13/463). Indeed, segmental duplication in the common carp genome might also cause disagreement between the physical and genetic linkage maps. For example, one of the anchored markers on a physical map contig might have been derived from a duplicated region and was then mapped to a duplicated region in a different linkage group. All other anchored markers were from single copy regions and could only be mapped onto one linkage group. The chimerical result reflects the real circumstances and may not be treated as errors in the physical map or genetic linkage mapping. Therefore, the error rate of the common carp physical map could be less than the estimated 3%.

### Comparative Analysis between Zebrafish and Common Carp Genomes

**Figure 2:**
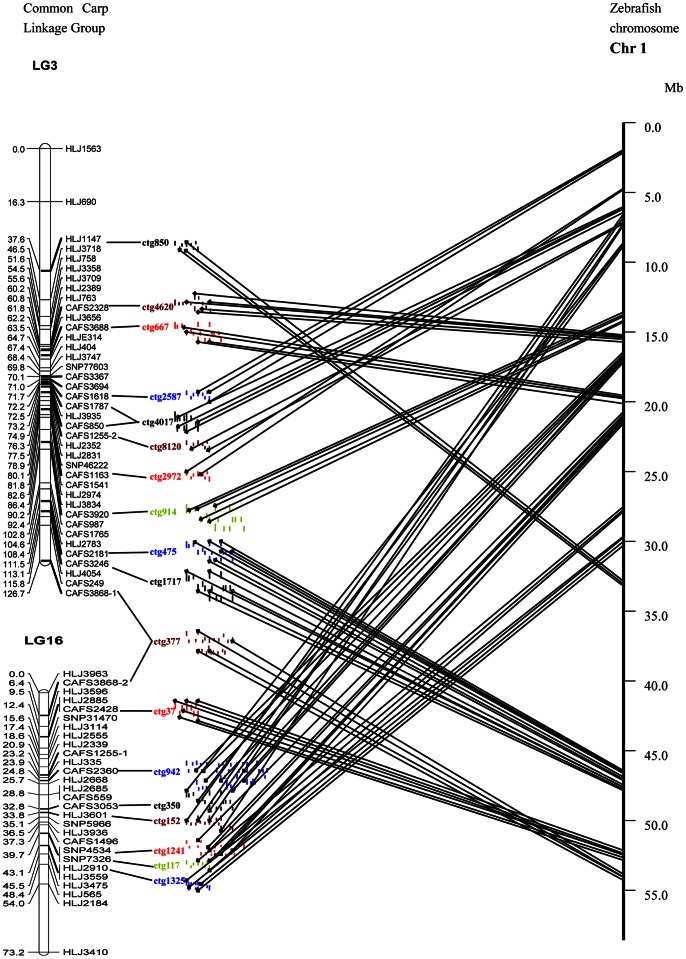
Conserved regions of synteny between the common carp linkage groups and zebrafish chromosome 1. Conserved syntenic regions were established by genetic linkage mapping of BAC contig-associated microsatellites and SNP markers. Bars on the left side are LG3 and LG16 from common carp and distances between markers are shown in cM. Contig-associated marker names (e.g., CAFS3688) and contig names (e.g., ctg667) are connected by a short thick line, demonstrating the anchor points of the integrated map. The assembled short lines represent the physical map contigs, and each contig and the contig name are marked by different colors. The small black diamond icons on each contig represent the BESs, which are connected to their homologous points on zebrafish chromosome 1 using solid lines.

Common carp has 100 chromosomes (2n) [48], [49], which is double of that of diploid cyprinids, such as the zebrafish. It had been shown that common carp have experienced an additional round of whole genome duplication (WGD) that doubled the chromosome number [9], [50]. Comparative analysis of the common carp genetic linkage map and zebrafish genome revealed a two-to-one relationship between common carp and zebrafish chromosomes [32]. Our integrated map updated the genetic linkage map of common carp and almost doubled the genetic markers, integrated over 498.75 Mb of the common carp genome and anchored 15,217 BAC clones and 19,681 BAC-end sequences to the genetic linkage map, which allowed us to further explore duplication in the common carp genome by comparison with the zebrafish genome. We aligned the sequences of all markers to the zebrafish reference genome (zv9) using BLASTn and successfully mapped 597 markers on 25 chromosomes, which increased the anchor points between the two genomes (Table S6). The results showed that two linkage groups of common carp were homologs with one particular chromosome of zebrafish. There was no exception in all 50 LGs of common carp, which confirmed the two-to-one homologous relationship of common carp and zebrafish chromosomes. Previous comparative analysis between genomes of zebrafish and common carp using large numbers of BESs resulted in a significant number of microsyntenies between the two genomes based on mate-paired BESs [21]. The physical map and integrated map provide a framework to map these microsyntenies on both common carp and zebrafish genomes. From this, we collected all 19,506 BESs from integrated contigs and performed a BLASTn search against the zebrafish genome, which revealed that 13,449 BESs had significant hits to zebrafish chromosomes with e-value cutoff of e-5 (Table S7). A vast majority of these BESs were mapped around anchoring points (SSR or SNP markers) on the integrated map. Therefore, microsyntenies based on mate-paired BESs were localized and merged as syntenies in these regions. We identified a total of 442 physical map contigs that formed syntenies between the two genomes. Chromosome-based macrosyntenies were further constructed between linkage groups of common carp and chromosomes of zebrafish, providing more details into the genome duplication of common carp and structural variation between the two genomes. For example, a total of 224 BESs anchored on LG 3 and LG 16 were mapped to the zebrafish genome with significant hits and we further mapped 18 physical map contigs on chromosome 1. Chromosome-scale syntenies were thus constructed between LG 3/16 of common carp and chromosome 1 of zebrafish (Figure 2). Other macrosyntenies between common carp and zebrafish genomes are shown in Figure S2. These macrosyntenies between common carp and zebrafish genomes clearly illustrated and comfirmed the common carp-specific WGD through comparative analysis. We also observed that some chromosomes in common carp were aligned to partial chromosomes in zebrafish, for instance, LG 17 was only aligned to 3/4 of chromosome 9 of zebrafish, LG 32 was only aligned to 1/2 of chromosome 22 of zebrafish, suggesting that segmental loss may have occurred after the latest WGD event in common carp.

Additional evidence of WGD came from the analysis of duplicated microsatellite markers. Thirteen primer pairs amplified double products that mapped to two different genomic loci. Both loci were genotyped and mapped successfully onto the genetic linkage map. All duplicated markers were treated as two markers during linkage analysis and mapping. For example, markers derived from CAFS2910 amplification were named as CAFS2910-1 and CAFS2910-2. All duplicated markers are listed in Table 4. Eight of the 13 duplicated markers (CAFS2910, CAFS1255, CAFS2152, CAFS642, CAFS3267, CAFS3617, CAFS3868 and CAFS3097) were mapped on two paired LGs, strengthening our presumption of the common carp-specific WGD event. The remaining five duplicated markers (CAFS671, CAFS2444, CAFS3169, CAFS1913 and CAFS3906) were mapped on five single LGs, which might have stemmed from segmental duplications in the chromosome rather than WGD.

**Table 4:** Duplicated markers on linkage groups of common carp gave evidence of common carp specific whole genome duplication.

Marker ID	Linkage group	Orthologous chromosome of zebrafish
CAFS1255-1	LG16	chr1
CAFS1255-2	LG3	chr1
CAFS3868-1	LG3	chr1
CAFS3868-2	LG16	chr1
CAFS2910-1	LG19	chr11
CAFS2910-2	LG50	chr11
CAFS642-1	LG36	chr13
CAFS642-2	LG43	chr13
CAFS3267-1	LG36	chr13
CAFS3267-2	LG43	chr13
CAFS3617-1	LG8	chr15
CAFS3617-2	LG9	chr15
CAFS3169-1	LG8	chr15
CAFS3169-2	LG8	chr15
CAFS2152-1	LG29	chr18
CAFS2152-2	LG7	chr18
CAFS3097-1	LG7	chr18
CAFS3097-2	LG29	chr18
CAFS1913-1	LG32	chr22
CAFS1913-2	LG32	chr22
CAFS2444-1	LG11	chr3
CAFS2444-2	LG11	chr3
CAFS671-1	LG30	chr7
CAFS671-2	LG30	chr7
CAFS3906-1	LG38	chr8
CAFS3906-2	LG38	chr8

### Conclusions

The integrated physical and genetic map of the common carp genome was constructed by mapping BAC-anchored markers to the genetic linkage map. An integrated map was composed of 1,209 genetic markers on 50 linkage groups. A total of 463 physical map contigs and 88 single BACs were anchored to the genetic linkage map, covering 498.75 Mb (corresponding to ca. 30% of the genome). Comparative analysis between the zebrafish and common carp genomes was performed using the integrated map and BES resources of common carp. The integrated map provides a tool for both genetic and genomic studies of common carp, which will help us understand the genome architecture of common carp and facilitate fine mapping and positional cloning of economically important traits for genetic improvement and modification.

## Methods

### Ethics Statement

This study was approved by the Animal Care and Use committee in the Centre for Applied Aquatic Genomics at Chinese Academy of Fishery Sciences.

### Available Physical Map and Genetic Linkage Map

The BAC library of common carp is composed of 92,160 BAC clones [20]. Over 40,000 BAC clones were previously sequenced from both ends that generated 65,720 cleaned BAC end sequences [21]. A physical map was constructed based on the same BAC library by assembling a total of 67,493 BAC clones into 3,696 contigs (http://genomics.cafs.ac.cn/fpc/WebAGCoL/Carp/WebFPC/) [22]. A recently constructed consensus genetic linkage map of common carp, comprising 617 markers served as a framework for map integration [32].

### Mapping Family

For map integration, a mapping family used for previous genetic linkage mapping [32] was used. Briefly, an F1 generation was constructed by crossing a female and a male common carp of the Songpu strain in the hatchery of Heilongjiang Fishery Research Institute. Fertilized eggs were brought to the hatchery and cultured in a one-ton tank. At 195 days post hatch (dph), the fry were transferred to 15 m^3^ rectangular tanks with continuous flow of water. At 300 dph, 190 F1 progeny were randomly selected from which blood samples (0.5 ml to 1 ml) and genomic DNA were obtained using the QIAamp DNA Blood Midi Kit (QIAGEN, Shanghai, China) for genotyping. A total of 107 progeny were used for genotyping and genetic linkage mapping.

### Development of Contig-anchored Microsatellite Markers

Physical map contigs were sorted by contig size and BAC end sequences were selected from contigs longer than 1 Mb. BES reads harboring microsatellite motifs with at least 50 bp flanking regions on either side were selected for PCR primer design with Primer3 software [51]. All primers were designed with optimal annealing temperatures ranging from 55°C to 60°C and product sizes ranging from 150 bp to 500 bp. To develop markers, at least two microsatellite loci were selected for marker development from each contig when sufficient contig-anchored microsatellites were available.

### Microsatellite Genotyping

A tailed primer protocol [52], [53] with the following conditions was used to amplify microsatellite alleles: 1× PCR buffer, 0.15 mM MgCl_2_, 0.2 mM of each the dNTP, 0.15 pmol upper PCR primer, 6 pmol lower PCR primer, 0.15 pmol labeled primer, 0.5 units of DNA Taq polymerase (Fermentas, Glen Burnie, MA, USA) and 20 ng genomic DNA in a reaction volume of 15 µl. Amplifications were performed on an AB 9700 thermocycler (Life technologies, Foster City, CA) with the following cycling conditions: an initial denaturation at 94°C for 5 min, then 30 cycles consisting of 94°C for 30 s, 56°C for 45 s and 72°C for 45 s, and 10 cycles consisting of 94°C for 30 s, 53°C for 45 s and 72°C for 45 s, followed by a final extension at 72°C for 10 min. PCR products were analyzed on a AB 3130×L Genetic Analyzer (Life Technologies) with a LIZ-500 size standard (Life Technologies) and genotyped with GeneMapper 4.0 software (Life Technologies). For convenience and efficiency, we selected 107 offspring and the parents for genotyping. Briefly, 2 parents and 94 offspring samples were placed on one 96-well plate as “large population”. Another 13 offspring samples, 1 positive control, 1 negative control and 1 blank control were placed on 16-well stripes as “small population”. New primers were first tested on the small population for screening potential polymorphic markers. Only those polymorphic markers were then genotyped in the large population.

### SNP Genotyping

The SNP genotypes were provided by colleagues that are currently working on high-density genetic mapping on the same mapping panel using RAD technology [47]. The flanking sequences of all SNP loci were compared with BESs on the physical map using BLASTn with a cutoff of e-5. Those SNPs with significant similarity to BESs were collected for our mapping integration.

### Aligning Markers to BAC End Sequences

Pre-existing microsatellites and SNP markers were mapped on the genetic linkage map by aligning their sequences in the BES database using the BLAST program. A minimum alignment length larger than 150 bp was set as the threshold for microsatellites and 100 bp was set for SNPs with sequence identity greater than 95%.

### Linkage Analysis

Genetic linkage maps were constructed using the software JoinMap version 4.0 (Kyazma, Wageningen, Netherlands). All genotype information from polymorphic markers on the mapping panel of 107 F1 siblings was used for linkage analysis. Before map construction, a “locus genotype frequency” function was used for a chi-square test of each marker to exclude several partial separation markers from the analysis. Default significance levels from 5.0 LOD to 15.0 LOD in steps of 1.0 were used. A mapping algorithm was selected as regression mapping and the maximum recombination rate was set at 0.4. Map distances were calculated using Kosambi’s mapping function.

### Comparative Analysis

To perform homologous analysis and identify regions of synteny between the integrated map of common carp and zebrafish genomes, all sequences on the integrated map of common carp with repeat and transposon sequences were masked, and then searched against the zebrafish genome assembly 9 (zv9) using BLASTn with an e-value cutoff of e-5. Top hits were used for further analysis.

## Supporting Information

Figure S1 **All 50 linkage groups of common carp integration map.**
(DOC)

Figure S2 **Additional macrosyntenies between common carp and zebrafish genomes.**
(PDF)

Figure S3 **Flowchart illustrates all markers sources for map integrations.**
(PPT)

Table S1 **Newly developed microsatellites were based on these 230 large physical map contigs.**
(XLS)

Table S2 **All information on BAC-anchored microsatellites.**
(XLS)

Table S3 **Information on SNP markers associated with BES.**
(XLS)

Table S4 **All genetic markers and their genotypes in a mapping family.**
(XLS)

Table S5 **Integrated contigs and single BAC clones on the integrated map.**
(XLS)

Table S6 **Putative conserved syntenies between common carp and zebrafish genomes.**
(XLS)

Table S7 **The BLASTn results of contigs on the integrated map of the common carp against zebrafish genome.**
(XLS)
