# Supplementary material for: A Dense Genetic Linkage Map for Common Carp and Its Integration with a BAC-Based Physical Map
Source: PLoS One. 2013 May 21;8(5):e63928. doi: 10.1371/journal.pone.0063928 (PMC3660343; doi:10.1371/journal.pone.0063928)
Supplement: Figure S2 — Additional macrosyntenies between common carp and zebrafish genomes. (PDF) [file pone.0063928.s002.pdf]

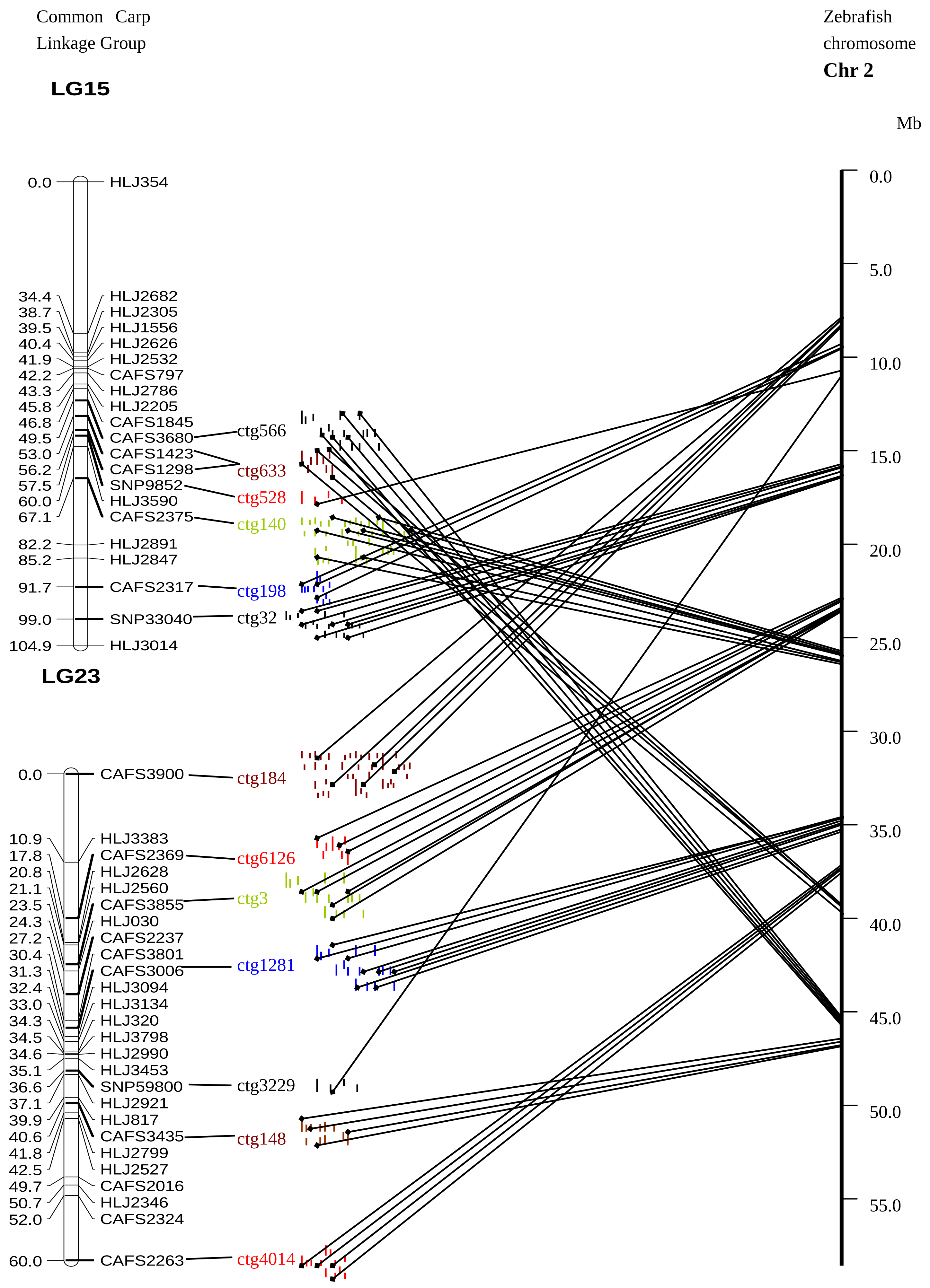
Common Carp
Linkage Group
LG15
Zebrafish
chromosome
Chr 2
Mb
0.0 HLJ354
34.4 HLJ2682
38.7 HLJ2305
39.5 HLJ1556
40.4 HLJ2626
41.9 HLJ2532
42.2 CAFS797
43.3 HLJ2786
45.8 HLJ2205
46.8 CAFS1845
49.5 CAFS3680
53.0 CAFS1423
56.2 CAFS1298
57.5 SNP9852
60.0 HLJ3590
67.1 CAFS2375
82.2 HLJ2891
85.2 HLJ2847
91.7 CAFS2317
99.0 SNP33040
104.9 HLJ3014
LG23
0.0 CAFS3900
10.9 HLJ3383
17.8 CAFS2369
20.8 HLJ2628
21.1 HLJ2560
23.5 CAFS3855
24.3 HLJ030
27.2 CAFS2237
30.4 CAFS3801
31.3 CAFS3006
32.4 HLJ3094
33.0 HLJ3134
34.3 HLJ320
34.5 HLJ3798
34.6 HLJ2990
35.1 HLJ3453
36.6 SNP59800
37.1 HLJ2921
39.9 HLJ817
40.6 CAFS3435
41.8 HLJ2799
42.5 HLJ2527
49.7 CAFS2016
50.7 HLJ2346
52.0 CAFS2324
60.0 CAFS2263
ctg566
ctg633
ctg528
ctg140
ctg198
ctg32
ctg184
ctg6126
ctg3
ctg1281
ctg3229
ctg148
ctg4014
0.0
5.0
10.0
15.0
20.0
25.0
30.0
35.0
40.0
45.0
50.0
55.0

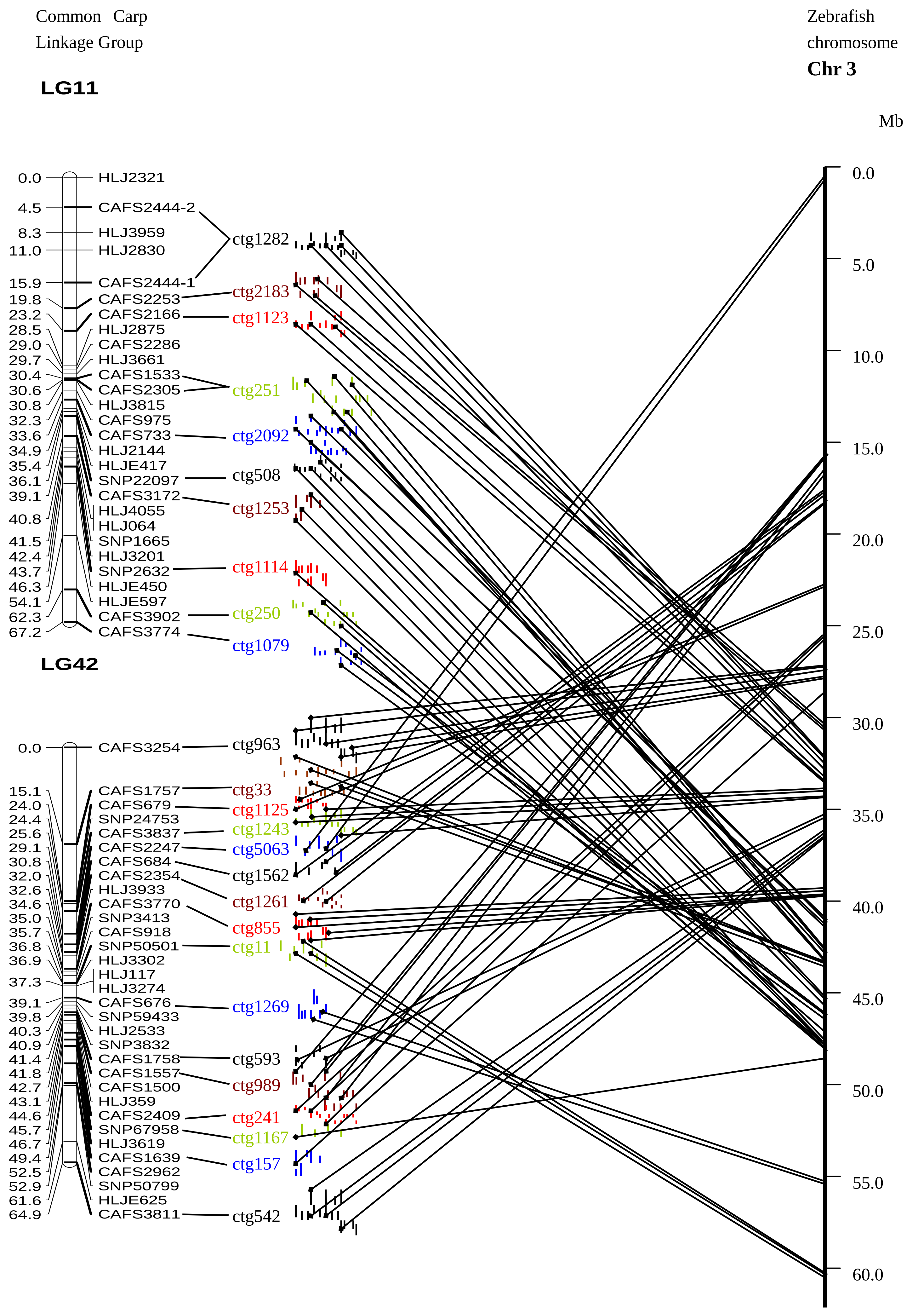

Common Carp
Linkage Group
LG11
Zebrafish
chromosome
Chr 3
Mb
0.0 HLJ2321
4.5 CAFS2444-2
8.3 HLJ3959
11.0 HLJ2830
15.9 CAFS2444-1
19.8 CAFS2253
23.2 CAFS2166
28.5 HLJ2875
29.0 CAFS2286
29.7 HLJ3661
30.4 CAFS1533
30.6 CAFS2305
30.8 HLJ3815
32.3 CAFS975
33.6 CAFS733
34.9 HLJ2144
35.4 HLJE417
36.1 SNP22097
39.1 CAFS3172
40.8 HLJ4055
HLJ064
41.5 SNP1665
42.4 HLJ3201
43.7 SNP2632
46.3 HLJE450
54.1 HLJE597
62.3 CAFS3902
67.2 CAFS3774
ctg1282
ctg2183
ctg1123
ctg251
ctg2092
ctg508
ctg1253
ctg1114
ctg250
ctg1079
LG42
0.0 CAFS3254
15.1 CAFS1757
24.0 CAFS679
24.4 SNP24753
25.6 CAFS3837
29.1 CAFS2247
30.8 CAFS684
32.0 CAFS2354
32.6 HLJ3933
34.6 CAFS3770
35.0 SNP3413
35.7 CAFS918
36.8 SNP50501
36.9 HLJ3302
37.3 HLJ117
HLJ3274
39.1 CAFS676
39.8 SNP59433
40.3 HLJ2533
40.9 SNP3832
41.4 CAFS1758
41.8 CAFS1557
42.7 CAFS1500
43.1 HLJ359
44.6 CAFS2409
45.7 SNP67958
46.7 HLJ3619
49.4 CAFS1639
52.5 CAFS2962
52.9 SNP50799
61.6 HLJE625
64.9 CAFS3811
ctg963
ctg33
ctg1125
ctg1243
ctg5063
ctg1562
ctg1261
ctg855
ctg11
ctg1269
ctg593
ctg989
ctg241
ctg1167
ctg157
ctg542
0.0
5.0
10.0
15.0
20.0
25.0
30.0
35.0
40.0
45.0
50.0
55.0
60.0

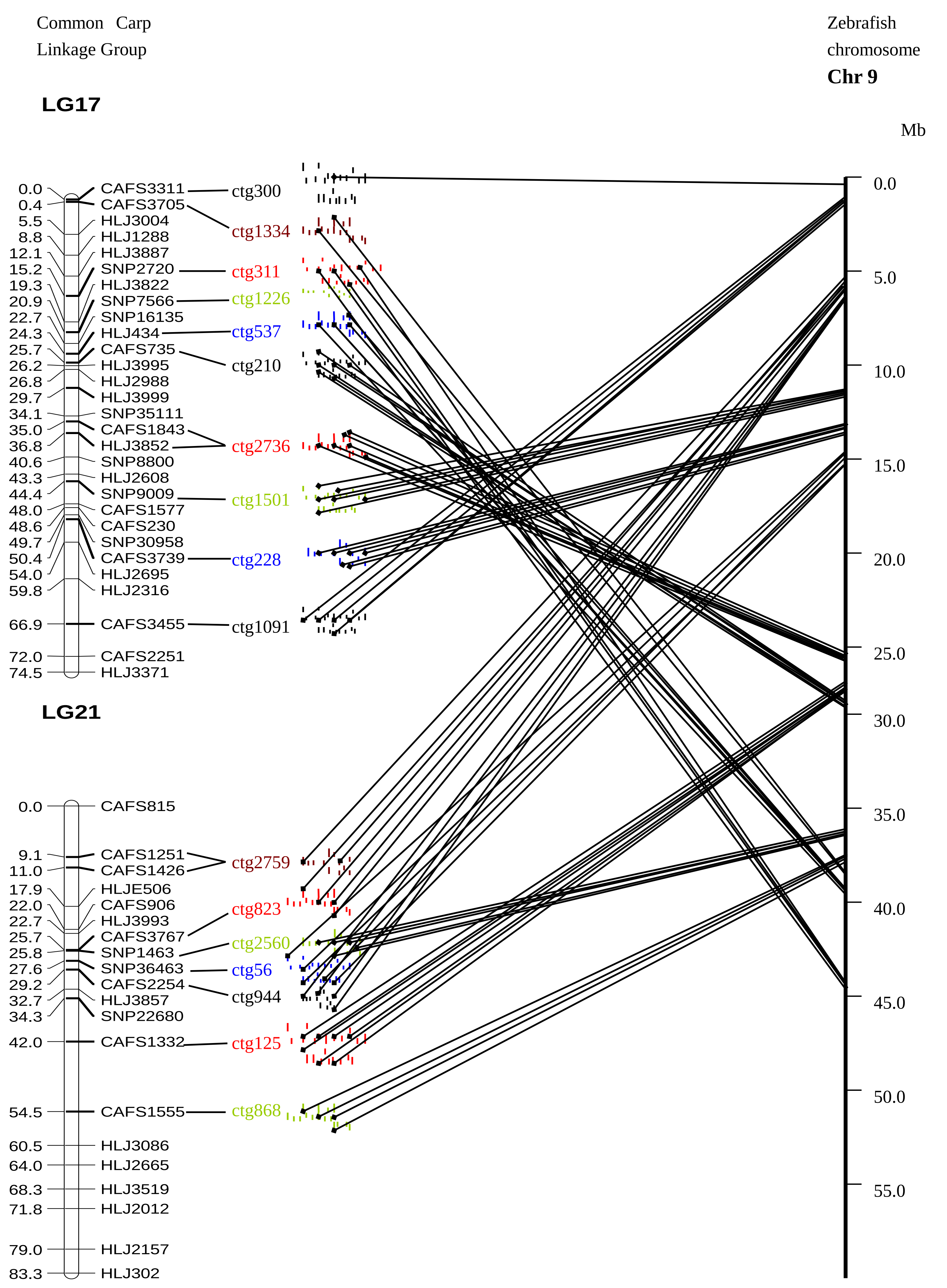

Common Carp
Linkage Group
Zebrafish
chromosome
Chr 9
LG17
Mb
0.0 CAFS3311 ctg300
0.4 CAFS3705 ctg1334
5.5 HLJ3004
8.8 HLJ1288
12.1 HLJ3887
15.2 SNP2720 ctg311
19.3 HLJ3822
20.9 SNP7566 ctg1226
22.7 SNP16135
24.3 HLJ434 ctg537
25.7 CAFS735
26.2 HLJ3995 ctg210
26.8 HLJ2988
29.7 HLJ3999
34.1 SNP35111
35.0 CAFS1843
36.8 HLJ3852 ctg2736
40.6 SNP8800
43.3 HLJ2608
44.4 SNP9009 ctg1501
48.0 CAFS1577
48.6 CAFS230
49.7 SNP30958
50.4 CAFS3739 ctg228
54.0 HLJ2695
59.8 HLJ2316
66.9 CAFS3455 ctg1091
72.0 CAFS2251
74.5 HLJ3371
LG21
0.0 CAFS815
9.1 CAFS1251 ctg2759
11.0 CAFS1426
17.9 HLJE506
22.0 CAFS906
22.7 HLJ3993 ctg823
25.7 CAFS3767
25.8 SNP1463 ctg2560
27.6 SNP36463 ctg56
29.2 CAFS2254 ctg944
32.7 HLJ3857
34.3 SNP22680
42.0 CAFS1332 ctg125
54.5 CAFS1555 ctg868
60.5 HLJ3086
64.0 HLJ2665
68.3 HLJ3519
71.8 HLJ2012
79.0 HLJ2157
83.3 HLJ302
0.0
5.0
10.0
15.0
20.0
25.0
30.0
35.0
40.0
45.0
50.0
55.0

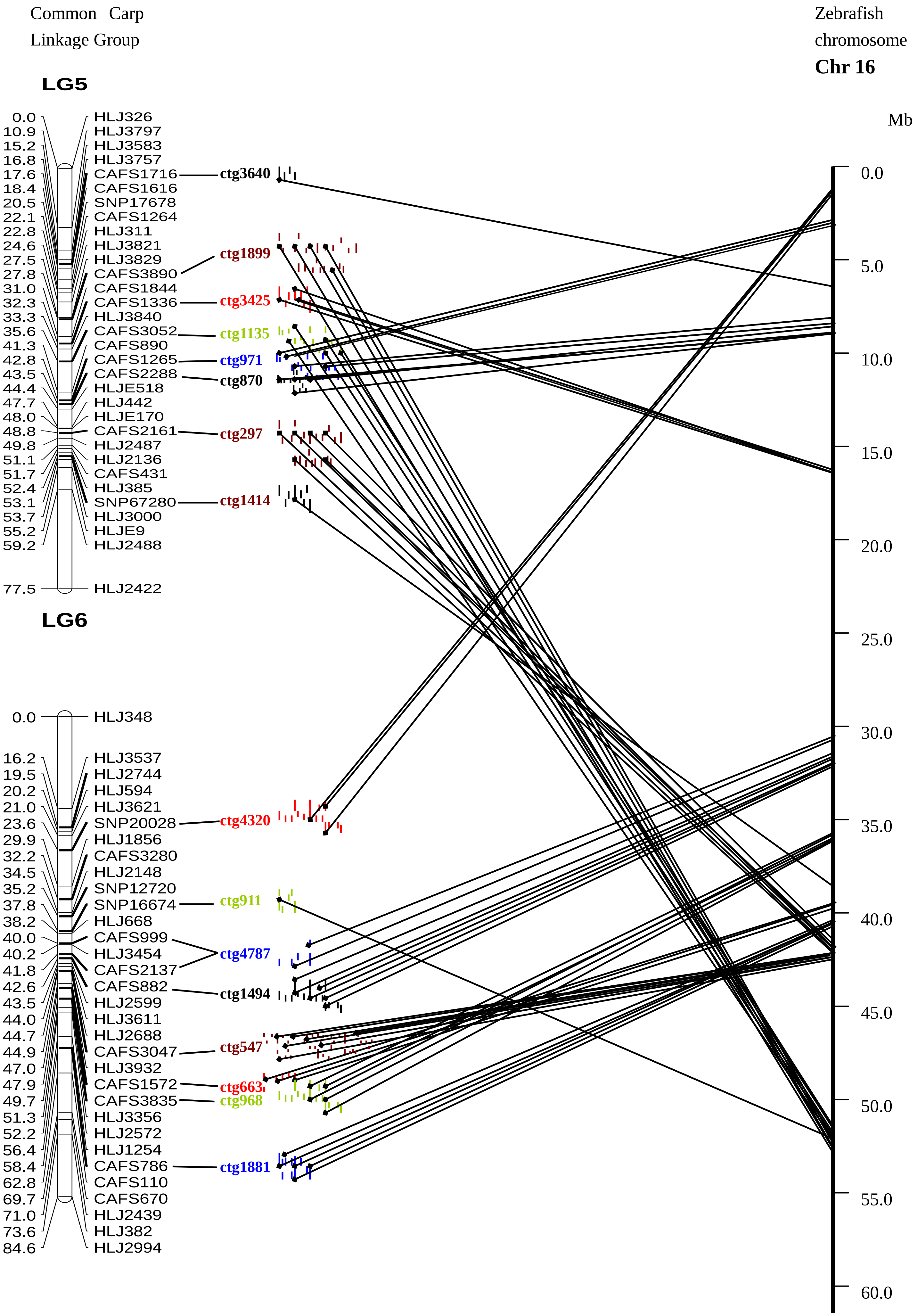

Common Carp
Linkage Group
Zebrafish
chromosome
Chr 16
LG5
Mb
0.0 HLJ326
10.9 HLJ3797
15.2 HLJ3583
16.8 HLJ3757
17.6 CAFS1716
18.4 CAFS1616
20.5 SNP17678
22.1 CAFS1264
22.8 HLJ311
24.6 HLJ3821
27.5 HLJ3829
27.8 CAFS3890
31.0 CAFS1844
32.3 CAFS1336
33.3 HLJ3840
35.6 CAFS3052
41.3 CAFS890
42.8 CAFS1265
43.5 CAFS2288
44.4 HLJE518
47.7 HLJ442
48.0 HLJE170
48.8 CAFS2161
49.8 HLJ2487
51.1 HLJ2136
51.7 CAFS431
52.4 HLJ385
53.1 SNP67280
53.7 HLJ3000
55.2 HLJE9
59.2 HLJ2488
77.5 HLJ2422
ctg3640
ctg1899
ctg3425
ctg1135
ctg971
ctg870
ctg297
ctg1414
LG6
0.0 HLJ348
16.2 HLJ3537
19.5 HLJ2744
20.2 HLJ594
21.0 HLJ3621
23.6 SNP20028
29.9 HLJ1856
32.2 CAFS3280
34.5 HLJ2148
35.2 SNP12720
37.8 SNP16674
38.2 HLJ668
40.0 CAFS999
40.2 HLJ3454
41.8 CAFS2137
42.6 CAFS882
43.5 HLJ2599
44.0 HLJ3611
44.7 HLJ2688
44.9 CAFS3047
47.0 HLJ3932
47.9 CAFS1572
49.7 CAFS3835
51.3 HLJ3356
52.2 HLJ2572
56.4 HLJ1254
58.4 CAFS786
62.8 CAFS110
69.7 CAFS670
71.0 HLJ2439
73.6 HLJ382
84.6 HLJ2994
ctg4320
ctg911
ctg4787
ctg1494
ctg547
ctg663
ctg968
ctg1881
0.0
5.0
10.0
15.0
20.0
25.0
30.0
35.0
40.0
45.0
50.0
55.0
60.0

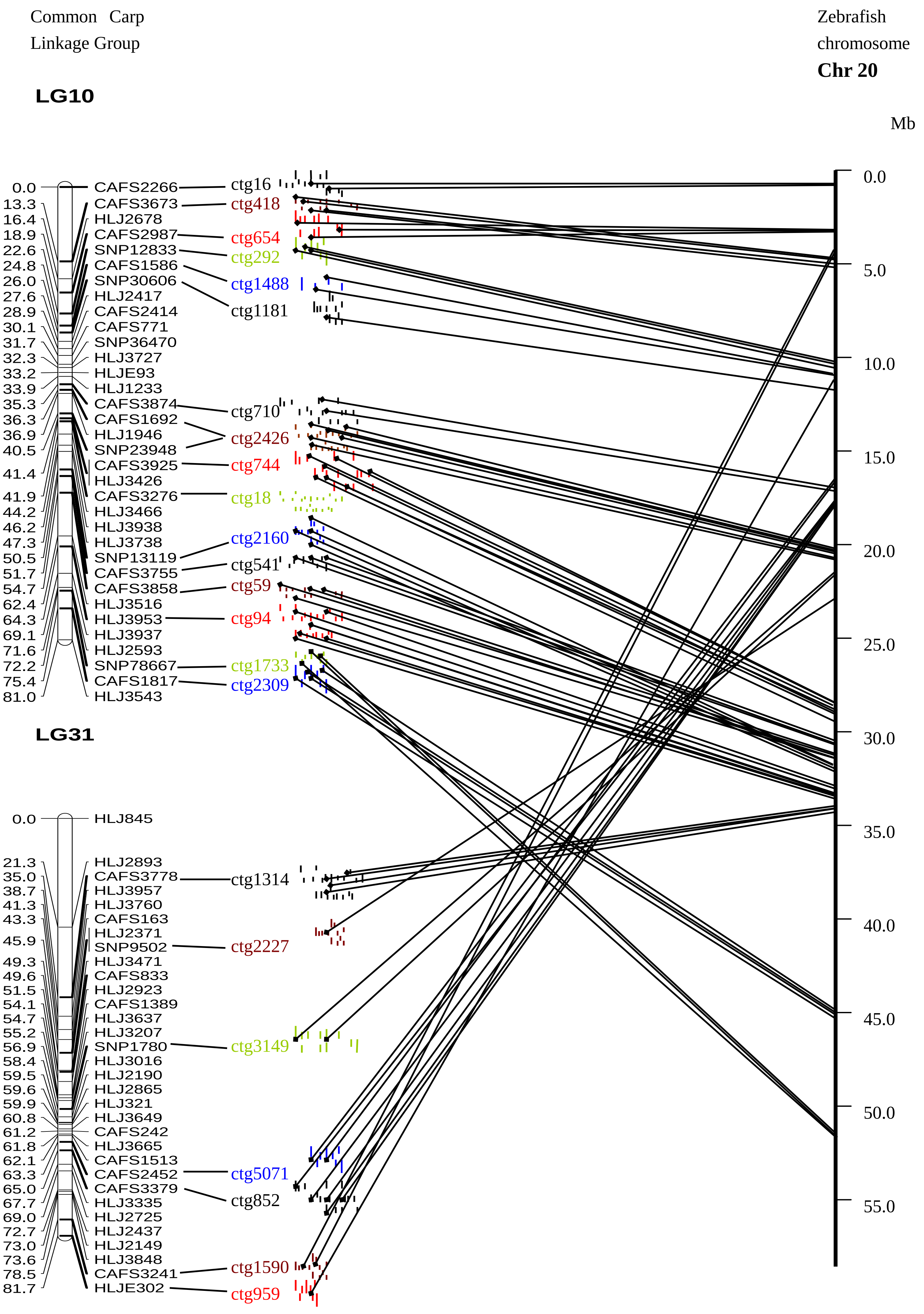

Common Carp
Linkage Group
Zebrafish
chromosome
Chr 20
Mb
LG10
0.0 CAFS2266 ctg16
13.3 CAFS3673 ctg418
16.4 HLJ2678
18.9 CAFS2987 ctg654
22.6 SNP12833 ctg292
24.8 CAFS1586 ctg1488
26.0 SNP30606 ctg1181
27.6 HLJ2417
28.9 CAFS2414
30.1 CAFS771
31.7 SNP36470
32.3 HLJ3727
33.2 HLJE93
33.9 HLJ1233
35.3 CAFS3874 ctg710
36.3 CAFS1692
36.9 HLJ1946 ctg2426
40.5 SNP23948
41.4 CAFS3925 ctg744
HLJ3426
41.9 CAFS3276 ctg18
44.2 HLJ3466
46.2 HLJ3938
47.3 HLJ3738 ctg2160
50.5 SNP13119
51.7 CAFS3755 ctg541
54.7 CAFS3858 ctg59
62.4 HLJ3516
64.3 HLJ3953 ctg94
69.1 HLJ3937
71.6 HLJ2593
72.2 SNP78667 ctg1733
75.4 CAFS1817 ctg2309
81.0 HLJ3543
LG31
0.0 HLJ845
21.3 HLJ2893
35.0 CAFS3778 ctg1314
38.7 HLJ3957
41.3 HLJ3760
43.3 CAFS163
45.9 HLJ2371
SNP9502 ctg2227
49.3 HLJ3471
49.6 CAFS833
51.5 HLJ2923
54.1 CAFS1389
54.7 HLJ3637
55.2 HLJ3207
56.9 SNP1780 ctg3149
58.4 HLJ3016
59.5 HLJ2190
59.6 HLJ2865
59.9 HLJ321
60.8 HLJ3649
61.2 CAFS242
61.8 HLJ3665
62.1 CAFS1513
63.3 CAFS2452 ctg5071
65.0 CAFS3379 ctg852
67.7 HLJ3335
69.0 HLJ2725
72.7 HLJ2437
73.0 HLJ2149
73.6 HLJ3848
78.5 CAFS3241 ctg1590
81.7 HLJE302 ctg959
0.0
5.0
10.0
15.0
20.0
25.0
30.0
35.0
40.0
45.0
50.0
55.0

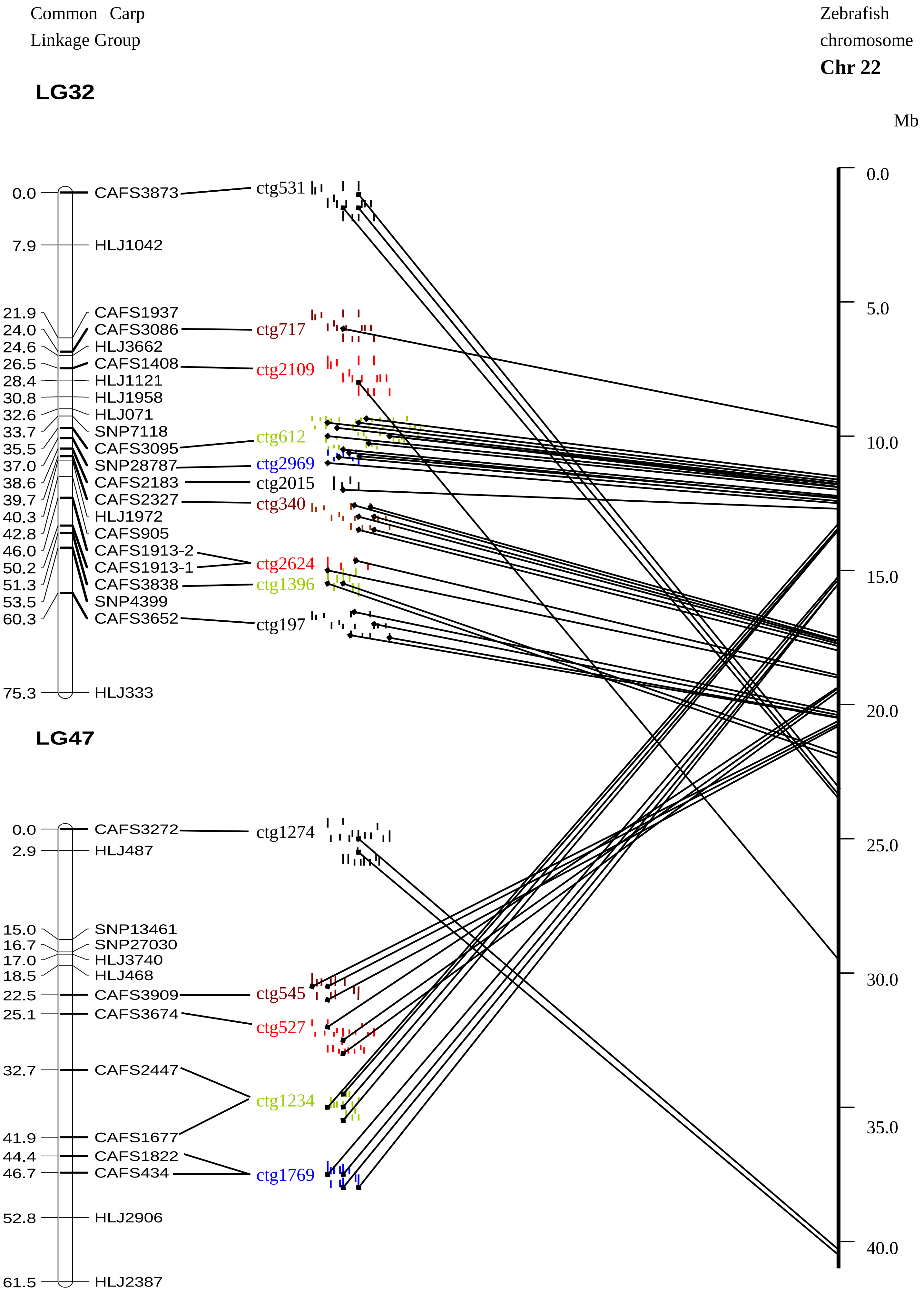

Common Carp
Linkage Group
LG32
Zebrafish
chromosome
Chr 22
Mb
0.0 CAFS3873
7.9 HLJ1042
21.9 CAFS1937
24.0 CAFS3086
24.6 HLJ3662
26.5 CAFS1408
28.4 HLJ1121
30.8 HLJ1958
32.6 HLJ071
33.7 SNP7118
35.5 CAFS3095
37.0 SNP28787
38.6 CAFS2183
39.7 CAFS2327
40.3 HLJ1972
42.8 CAFS905
46.0 CAFS1913-2
50.2 CAFS1913-1
51.3 CAFS3838
53.5 SNP4399
60.3 CAFS3652
75.3 HLJ333
ctg531
ctg717
ctg2109
ctg612
ctg2969
ctg2015
ctg340
ctg2624
ctg1396
ctg197
LG47
0.0 CAFS3272
2.9 HLJ487
15.0 SNP13461
16.7 SNP27030
17.0 HLJ3740
18.5 HLJ468
22.5 CAFS3909
25.1 CAFS3674
32.7 CAFS2447
41.9 CAFS1677
44.4 CAFS1822
46.7 CAFS434
52.8 HLJ2906
61.5 HLJ2387
ctg1274
ctg545
ctg527
ctg1234
ctg1769
0.0
5.0
10.0
15.0
20.0
25.0
30.0
35.0
40.0
