# Supplementary material for: A Dense Genetic Linkage Map for Common Carp and Its Integration with a BAC-Based Physical Map
Source: PLoS One. 2013 May 21;8(5):e63928. doi: 10.1371/journal.pone.0063928 (PMC3660343; doi:10.1371/journal.pone.0063928)
Supplement: Figure S3 — Flowchart illustrates all markers sources for map integrations. (PPT) [file pone.0063928.s003.ppt]

## Slide 1
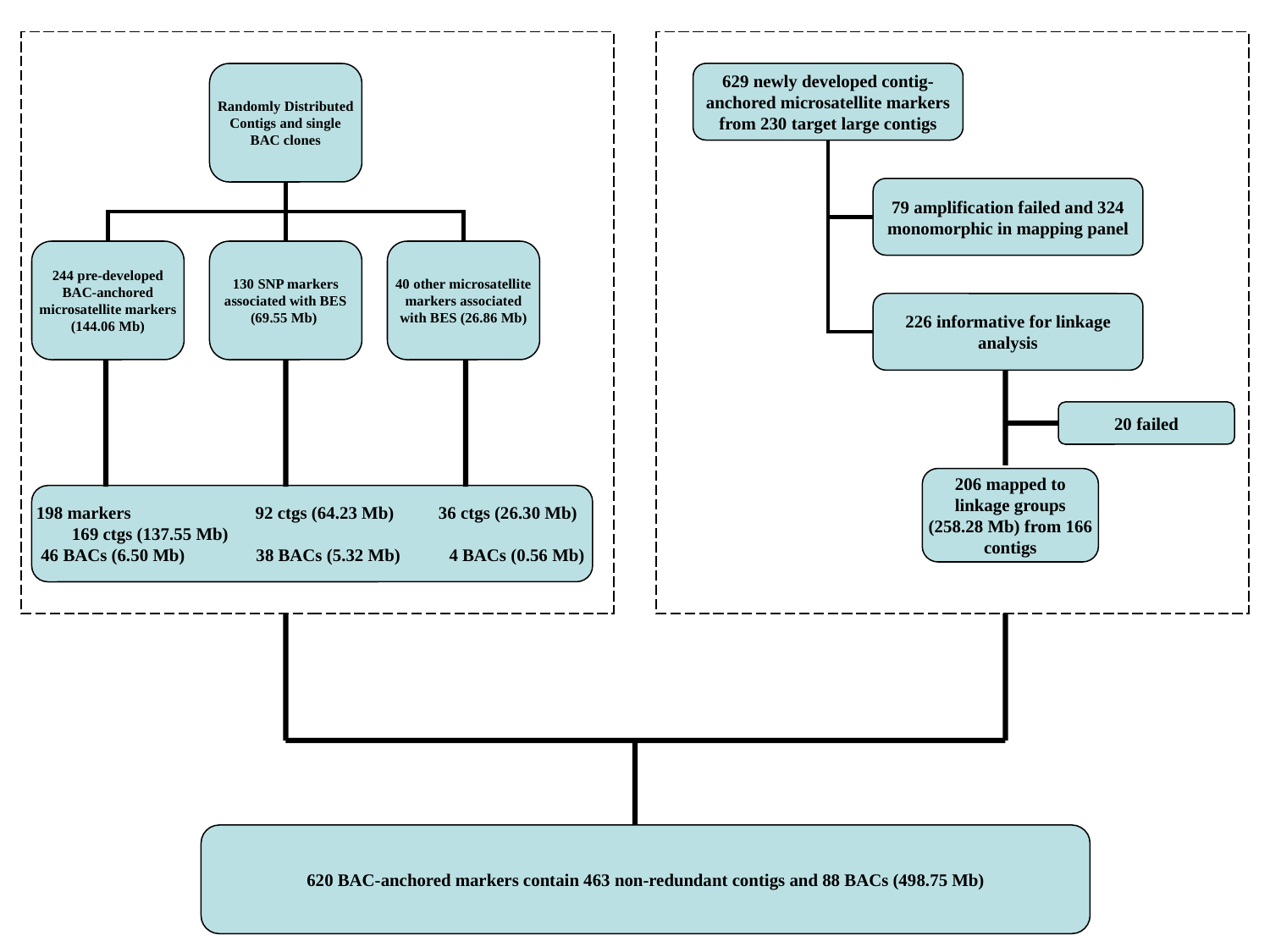

Randomly Distributed Contigs and single BAC clones
244 pre-developed BAC-anchored microsatellite markers (144.06 Mb)
130 SNP markers associated with BES (69.55 Mb)
40 other microsatellite markers associated with BES (26.86 Mb)
629 newly developed contig-anchored microsatellite markers from 230 target large contigs
79 amplification failed and 324 monomorphic in mapping panel
226 informative for linkage analysis
20 failed
206 mapped to linkage groups (258.28 Mb) from 166 contigs
198 markers 92 ctgs (64.23 Mb) 36 ctgs (26.30 Mb)
 169 ctgs (137.55 Mb)
 46 BACs (6.50 Mb) 38 BACs (5.32 Mb) 4 BACs (0.56 Mb)
620 BAC-anchored markers contain 463 non-redundant contigs and 88 BACs (498.75 Mb)
