# Supplementary material for: A Dense Genetic Linkage Map for Common Carp and Its Integration with a BAC-Based Physical Map
Source: PLoS One. 2013 May 21;8(5):e63928. doi: 10.1371/journal.pone.0063928 (PMC3660343; doi:10.1371/journal.pone.0063928)
Supplement: Supplementary file 3 [file pone.0063928.s001.doc]

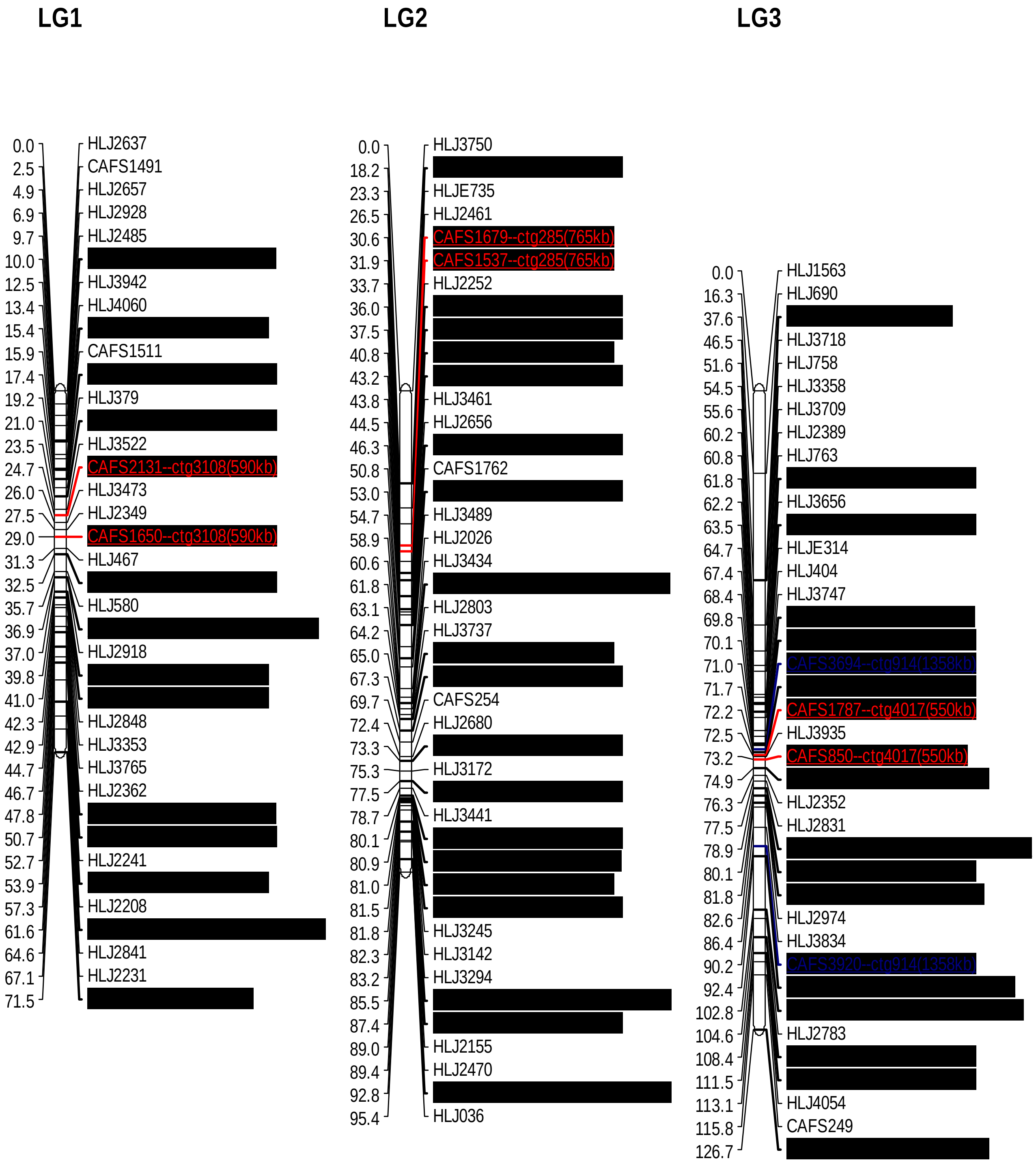
LG1
0.0 HLJ2637
2.5 CAFS1491
4.9 HLJ2657
6.9 HLJ2928
9.7 HLJ2485
10.0 [redacted]
12.5 HLJ3942
13.4 HLJ4060
15.4 [redacted]
15.9 CAFS1511
17.4 [redacted]
19.2 HLJ379
21.0 [redacted]
23.5 HLJ3522
24.7 CAFS2131--ctg3108(590kb)
26.0 HLJ3473
27.5 HLJ2349
29.0 CAFS1650--ctg3108(590kb)
31.3 HLJ467
32.5 [redacted]
35.7 HLJ580
36.9 [redacted]
37.0 HLJ2918
39.8 [redacted]
41.0 [redacted]
42.3 HLJ2848
42.9 HLJ3353
44.7 HLJ3765
46.7 HLJ2362
47.8 [redacted]
50.7 [redacted]
52.7 HLJ2241
53.9 [redacted]
57.3 HLJ2208
61.6 [redacted]
64.6 HLJ2841
67.1 HLJ2231
71.5 [redacted]
LG2
0.0 HLJ3750
18.2 [redacted]
23.3 HLJE735
26.5 HLJ2461
30.6 CAFS1679--ctg285(765kb)
31.9 CAFS1537--ctg285(765kb)
33.7 HLJ2252
36.0 [redacted]
37.5 [redacted]
40.8 [redacted]
43.2 [redacted]
43.8 HLJ3461
44.5 HLJ2656
46.3 [redacted]
50.8 CAFS1762
53.0 [redacted]
54.7 HLJ3489
58.9 HLJ2026
60.6 HLJ3434
61.8 [redacted]
63.1 HLJ2803
64.2 HLJ3737
65.0 [redacted]
67.3 [redacted]
69.7 CAFS254
72.4 HLJ2680
73.3 [redacted]
75.3 HLJ3172
77.5 [redacted]
78.7 HLJ3441
80.1 [redacted]
80.9 [redacted]
81.0 [redacted]
81.5 [redacted]
81.8 HLJ3245
82.3 HLJ3142
83.2 HLJ3294
85.5 [redacted]
87.4 [redacted]
89.0 HLJ2155
89.4 HLJ2470
92.8 [redacted]
95.4 HLJ036
LG3
0.0 HLJ1563
16.3 HLJ690
37.6 [redacted]
46.5 HLJ3718
51.6 HLJ758
54.5 HLJ3358
55.6 HLJ3709
60.2 HLJ2389
60.8 HLJ763
61.8 [redacted]
62.2 HLJ3656
63.5 [redacted]
64.7 HLJE314
67.4 HLJ404
68.4 HLJ3747
69.8 [redacted]
70.1 [redacted]
71.0 CAFS3694--ctg914(1358kb)
71.7 [redacted]
72.2 CAFS1787--ctg4017(550kb)
72.5 HLJ3935
73.2 CAFS850--ctg4017(550kb)
74.9 [redacted]
76.3 HLJ2352
77.5 HLJ2831
78.9 [redacted]
80.1 [redacted]
81.8 [redacted]
82.6 HLJ2974
86.4 HLJ3834
90.2 CAFS3920--ctg914(1358kb)
92.4 [redacted]
102.8 [redacted]
104.6 HLJ2783
108.4 [redacted]
111.5 [redacted]
113.1 HLJ4054
115.8 CAFS249
126.7 [redacted]

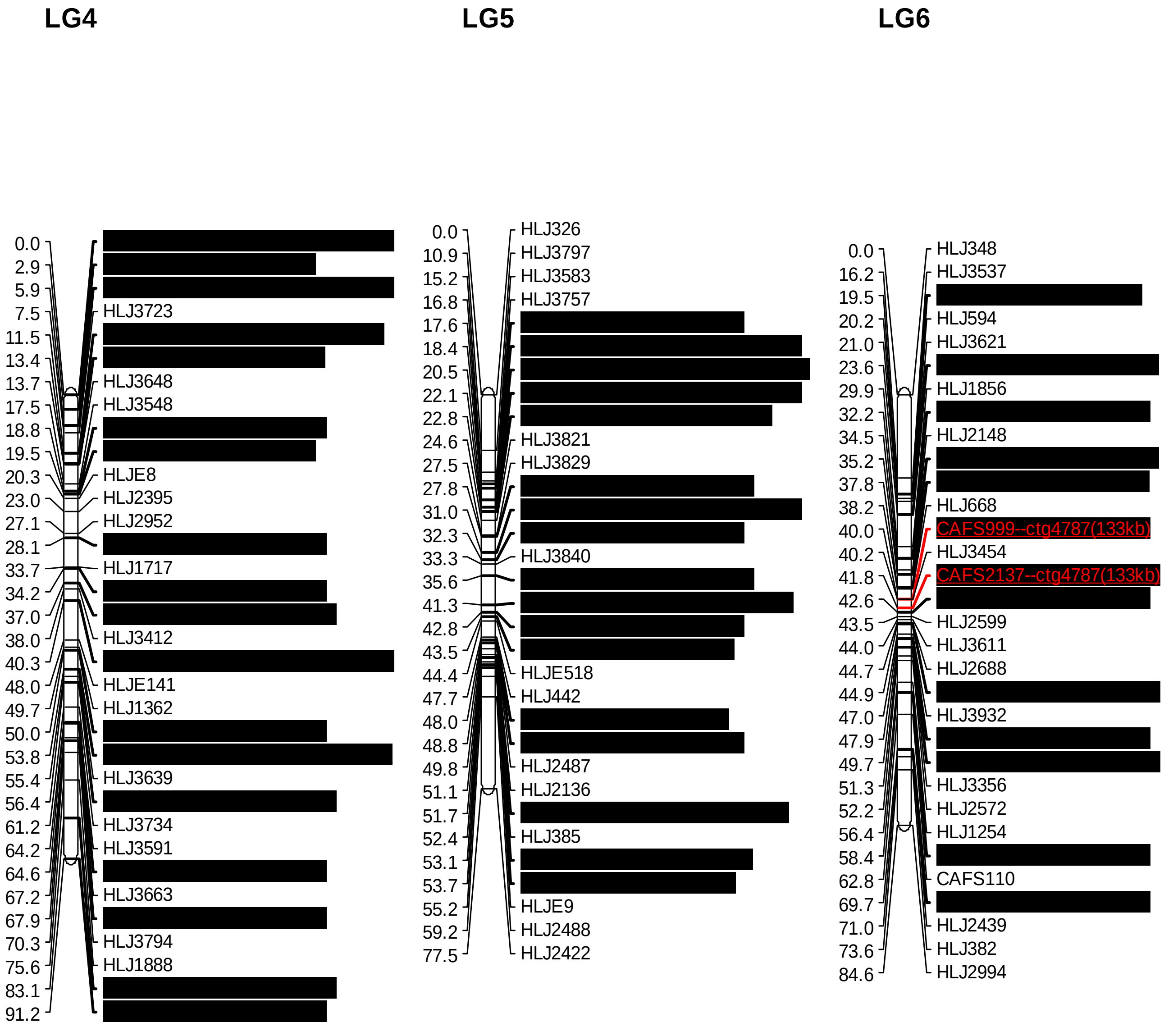
LG4
0.0 [redacted]
2.9 [redacted]
5.9 [redacted]
7.5 HLJ3723
11.5 [redacted]
13.4 [redacted]
13.7 HLJ3648
17.5 HLJ3548
18.8 [redacted]
19.5 [redacted]
20.3 HLJE8
23.0 HLJ2395
27.1 HLJ2952
28.1 [redacted]
33.7 HLJ1717
34.2 [redacted]
37.0 [redacted]
38.0 HLJ3412
40.3 [redacted]
48.0 HLJE141
49.7 HLJ1362
50.0 [redacted]
53.8 [redacted]
55.4 HLJ3639
56.4 [redacted]
61.2 HLJ3734
64.2 HLJ3591
64.6 [redacted]
67.2 HLJ3663
67.9 [redacted]
70.3 HLJ3794
75.6 HLJ1888
83.1 [redacted]
91.2 [redacted]
LG5
0.0 HLJ326
10.9 HLJ3797
15.2 HLJ3583
16.8 HLJ3757
17.6 [redacted]
18.4 [redacted]
20.5 [redacted]
22.1 [redacted]
22.8 [redacted]
24.6 HLJ3821
27.5 HLJ3829
27.8 [redacted]
31.0 [redacted]
32.3 [redacted]
33.3 HLJ3840
35.6 [redacted]
41.3 [redacted]
42.8 [redacted]
43.5 [redacted]
44.4 HLJE518
47.7 HLJ442
48.0 [redacted]
48.8 [redacted]
49.8 HLJ2487
51.1 HLJ2136
51.7 [redacted]
52.4 HLJ385
53.1 [redacted]
53.7 [redacted]
55.2 HLJE9
59.2 HLJ2488
77.5 HLJ2422
LG6
0.0 HLJ348
16.2 HLJ3537
19.5 [redacted]
20.2 HLJ594
21.0 HLJ3621
23.6 [redacted]
29.9 HLJ1856
32.2 [redacted]
34.5 HLJ2148
35.2 [redacted]
37.8 [redacted]
38.2 HLJ668
40.0 CAFS999--ctg4787(133kb)
40.2 HLJ3454
41.8 CAFS2137--ctg4787(133kb)
42.6 [redacted]
43.5 HLJ2599
44.0 HLJ3611
44.7 HLJ2688
44.9 [redacted]
47.0 HLJ3932
47.9 [redacted]
49.7 [redacted]
51.3 HLJ3356
52.2 HLJ2572
56.4 HLJ1254
58.4 [redacted]
62.8 CAFS110
69.7 [redacted]
71.0 HLJ2439
73.6 HLJ382
84.6 HLJ2994

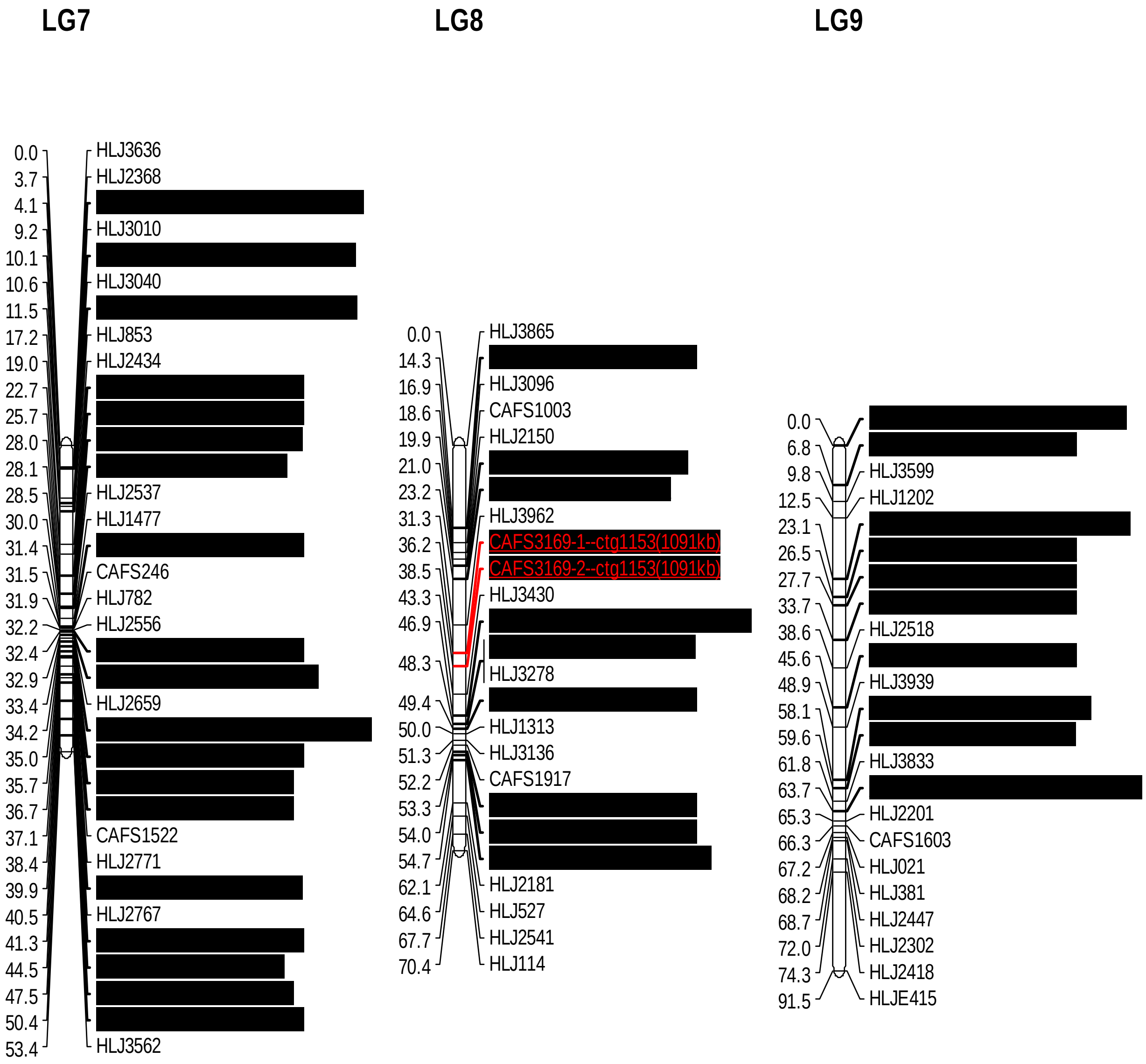
LG7
0.0
3.7
4.1
9.2
10.1
10.6
11.5
17.2
19.0
22.7
25.7
28.0
28.1
28.5
30.0
31.4
31.5
31.9
32.2
32.4
32.9
33.4
34.2
35.0
35.7
36.7
37.1
38.4
39.9
40.5
41.3
44.5
47.5
50.4
53.4
HLJ3636
HLJ2368
[redacted]
HLJ3010
[redacted]
HLJ3040
[redacted]
HLJ853
HLJ2434
[redacted]
HLJ2537
HLJ1477
[redacted]
CAFS246
HLJ782
HLJ2556
[redacted]
HLJ2659
[redacted]
CAFS1522
HLJ2771
[redacted]
HLJ2767
[redacted]
HLJ3562
LG8
0.0
14.3
16.9
18.6
19.9
21.0
23.2
31.3
36.2
38.5
43.3
46.9
48.3
49.4
50.0
51.3
52.2
53.3
54.0
54.7
62.1
64.6
67.7
70.4
HLJ3865
[redacted]
HLJ3096
CAFS1003
HLJ2150
[redacted]
HLJ3962
CAFS3169-1--ctg1153(1091kb)
CAFS3169-2--ctg1153(1091kb)
HLJ3430
[redacted]
HLJ3278
[redacted]
HLJ1313
HLJ3136
CAFS1917
[redacted]
HLJ2181
HLJ527
HLJ2541
HLJ114
LG9
0.0
6.8
9.8
12.5
23.1
26.5
27.7
33.7
38.6
45.6
48.9
58.1
59.6
61.8
63.7
65.3
66.3
67.2
68.2
68.7
72.0
74.3
91.5
[redacted]
HLJ3599
HLJ1202
[redacted]
HLJ2518
[redacted]
HLJ3939
[redacted]
HLJ3833
[redacted]
HLJ2201
CAFS1603
HLJ021
HLJ381
HLJ2447
HLJ2302
HLJ2418
HLJE415

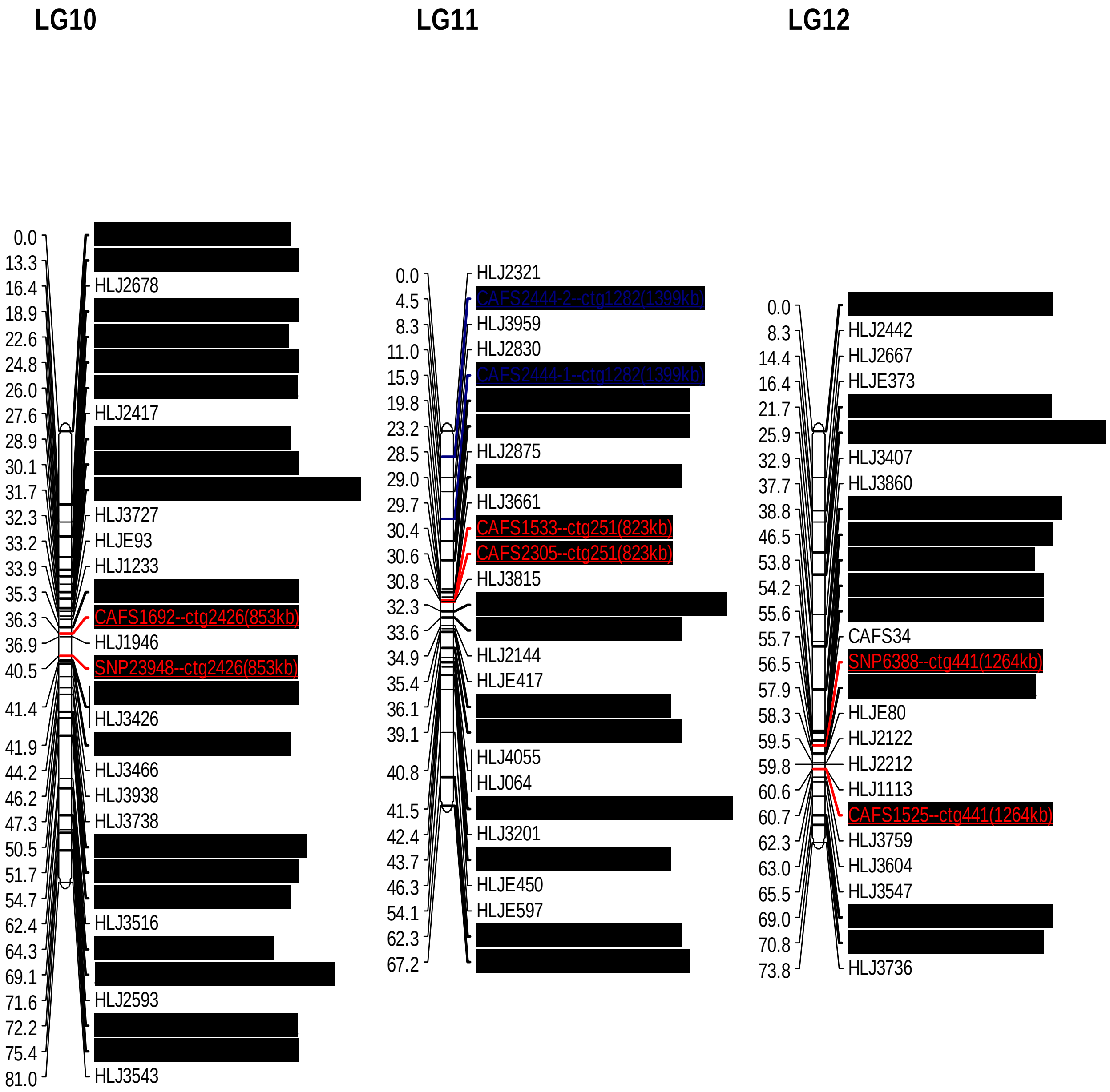
LG10
0.0 [redacted]
13.3 [redacted]
16.4 HLJ2678
18.9 [redacted]
22.6 [redacted]
24.8 [redacted]
26.0 [redacted]
27.6 HLJ2417
28.9 [redacted]
30.1 [redacted]
31.7 [redacted]
32.3 HLJ3727
33.2 HLJE93
33.9 HLJ1233
35.3 [redacted]
36.3 CAFS1692--ctg2426(853kb)
36.9 HLJ1946
40.5 SNP23948--ctg2426(853kb)
[redacted]
41.4 HLJ3426
41.9 [redacted]
44.2 HLJ3466
46.2 HLJ3938
47.3 HLJ3738
50.5 [redacted]
51.7 [redacted]
54.7 [redacted]
62.4 HLJ3516
64.3 [redacted]
69.1 [redacted]
71.6 HLJ2593
72.2 [redacted]
75.4 [redacted]
81.0 HLJ3543
LG11
0.0 HLJ2321
4.5 CAFS2444-2--ctg1282(1399kb)
8.3 HLJ3959
11.0 HLJ2830
15.9 CAFS2444-1--ctg1282(1399kb)
19.8 [redacted]
23.2 [redacted]
28.5 HLJ2875
29.0 [redacted]
29.7 HLJ3661
30.4 CAFS1533--ctg251(823kb)
30.6 CAFS2305--ctg251(823kb)
30.8 HLJ3815
32.3 [redacted]
33.6 [redacted]
34.9 HLJ2144
35.4 HLJE417
36.1 [redacted]
39.1 [redacted]
40.8 HLJ4055
HLJ064
41.5 [redacted]
42.4 HLJ3201
43.7 [redacted]
46.3 HLJE450
54.1 HLJE597
62.3 [redacted]
67.2 [redacted]
LG12
0.0 [redacted]
8.3 HLJ2442
14.4 HLJ2667
16.4 HLJE373
21.7 [redacted]
25.9 [redacted]
32.9 HLJ3407
37.7 HLJ3860
38.8 [redacted]
46.5 [redacted]
53.8 [redacted]
54.2 [redacted]
55.6 [redacted]
55.7 CAFS34
56.5 SNP6388--ctg441(1264kb)
57.9 [redacted]
58.3 HLJE80
59.5 HLJ2122
59.8 HLJ2212
60.6 HLJ1113
60.7 CAFS1525--ctg441(1264kb)
62.3 HLJ3759
63.0 HLJ3604
65.5 HLJ3547
69.0 [redacted]
70.8 [redacted]
73.8 HLJ3736

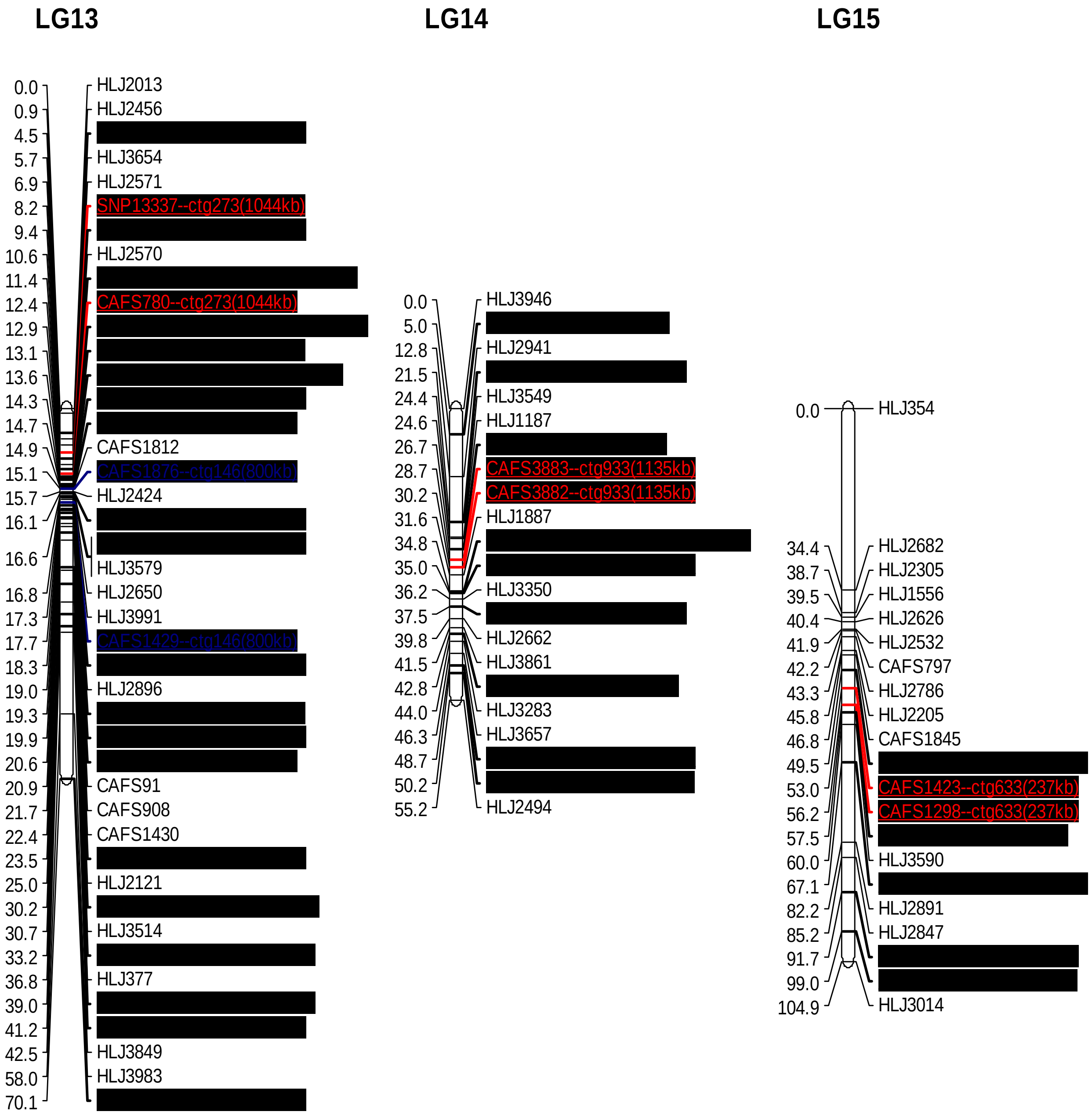

LG13
0.0 HLJ2013
0.9 HLJ2456
4.5 [redacted]
5.7 HLJ3654
6.9 HLJ2571
8.2 SNP13337--ctg273(1044kb)
9.4 [redacted]
10.6 HLJ2570
11.4 [redacted]
12.4 CAFS780--ctg273(1044kb)
12.9 [redacted]
13.1 [redacted]
13.6 [redacted]
14.3 [redacted]
14.7 [redacted]
14.9 CAFS1812
15.1 CAFS1876--ctg146(800kb)
15.7 HLJ2424
16.1 [redacted]
16.6 [redacted]
HLJ3579
16.8 HLJ2650
17.3 HLJ3991
17.7 CAFS1429--ctg146(800kb)
18.3 [redacted]
19.0 HLJ2896
19.3 [redacted]
19.9 [redacted]
20.6 [redacted]
20.9 CAFS91
21.7 CAFS908
22.4 CAFS1430
23.5 [redacted]
25.0 HLJ2121
30.2 [redacted]
30.7 HLJ3514
33.2 [redacted]
36.8 HLJ377
39.0 [redacted]
41.2 [redacted]
42.5 HLJ3849
58.0 HLJ3983
70.1 [redacted]
LG14
0.0 HLJ3946
5.0 [redacted]
12.8 HLJ2941
21.5 [redacted]
24.4 HLJ3549
24.6 HLJ1187
26.7 [redacted]
28.7 CAFS3883--ctg933(1135kb)
30.2 CAFS3882--ctg933(1135kb)
31.6 HLJ1887
34.8 [redacted]
35.0 [redacted]
36.2 HLJ3350
37.5 [redacted]
39.8 HLJ2662
41.5 HLJ3861
42.8 [redacted]
44.0 HLJ3283
46.3 HLJ3657
48.7 [redacted]
50.2 [redacted]
55.2 HLJ2494
LG15
0.0 HLJ354
34.4 HLJ2682
38.7 HLJ2305
39.5 HLJ1556
40.4 HLJ2626
41.9 HLJ2532
42.2 CAFS797
43.3 HLJ2786
45.8 HLJ2205
46.8 CAFS1845
49.5 [redacted]
53.0 CAFS1423--ctg633(237kb)
56.2 CAFS1298--ctg633(237kb)
57.5 [redacted]
60.0 HLJ3590
67.1 [redacted]
82.2 HLJ2891
85.2 HLJ2847
91.7 [redacted]
99.0 [redacted]
104.9 HLJ3014

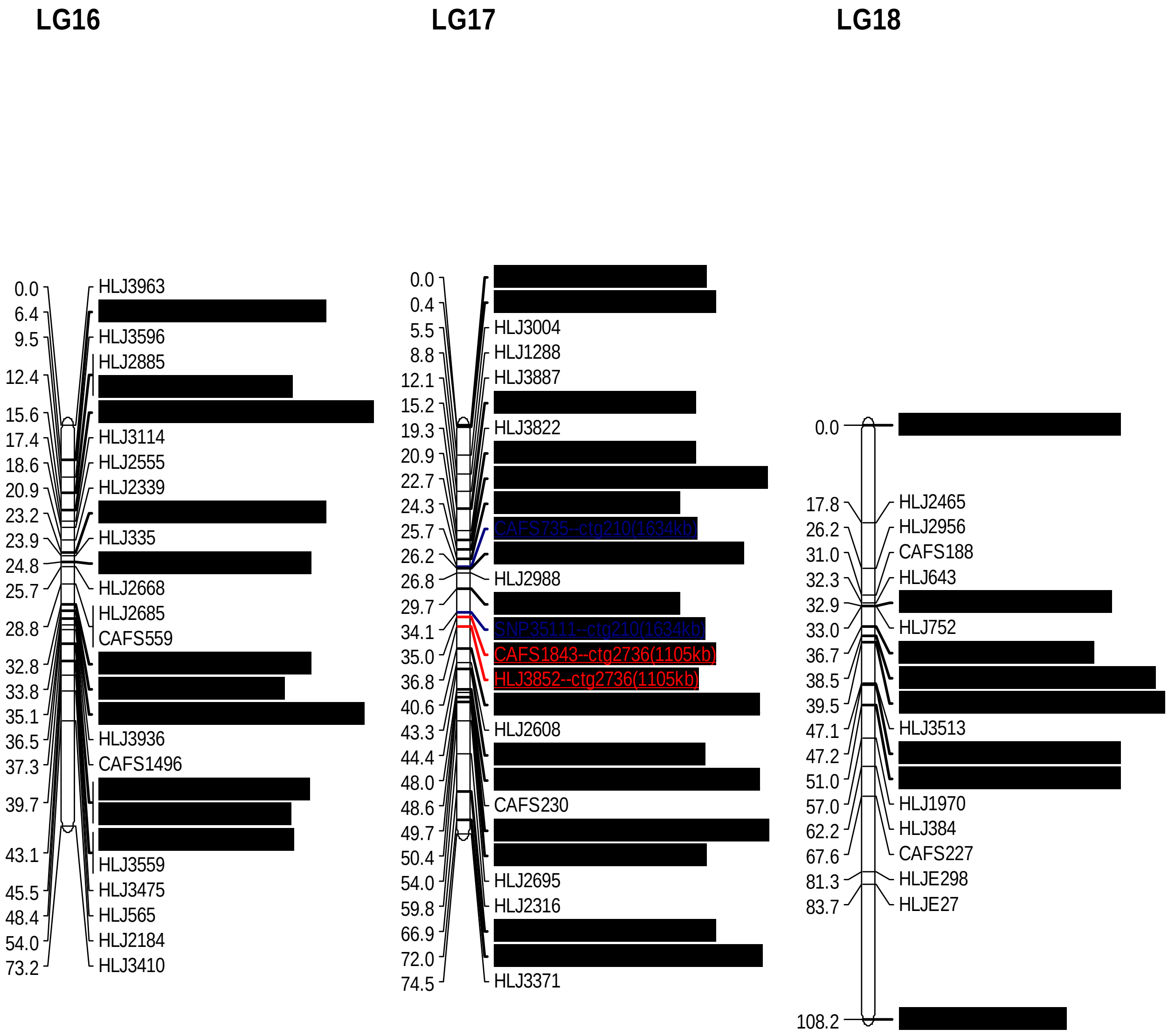
LG16
0.0 HLJ3963
6.4 [redacted]
9.5 HLJ3596
12.4 HLJ2885
15.6 [redacted]
17.4 HLJ3114
18.6 HLJ2555
20.9 HLJ2339
23.2 [redacted]
23.9 HLJ335
24.8 [redacted]
25.7 HLJ2668
28.8 HLJ2685
32.8 CAFS559
33.8 [redacted]
35.1
36.5 HLJ3936
37.3 CAFS1496
39.7 [redacted]
43.1 HLJ3559
45.5 HLJ3475
48.4 HLJ565
54.0 HLJ2184
73.2 HLJ3410
LG17
0.0 [redacted]
0.4
5.5 HLJ3004
8.8 HLJ1288
12.1 HLJ3887
15.2 [redacted]
19.3 HLJ3822
20.9 [redacted]
22.7
24.3
25.7 CAFS736--ctg210(1634kb)
26.2 [redacted]
26.8 HLJ2988
29.7 [redacted]
34.1 SNP35111--ctg210(1634kb)
35.0 CAFS1843--ctg2736(1105kb)
36.8 HLJ3852--ctg2736(1105kb)
40.6 [redacted]
43.3 HLJ2608
44.4 [redacted]
48.0
48.6 CAFS230
49.7 [redacted]
50.4
54.0 HLJ2695
59.8 HLJ2316
66.9 [redacted]
72.0
74.5 HLJ3371
LG18
0.0 [redacted]
17.8 HLJ2465
26.2 HLJ2956
31.0 CAFS188
32.3 HLJ643
32.9 [redacted]
33.0 HLJ752
36.7 [redacted]
38.5
39.5
47.1 HLJ3513
47.2 [redacted]
51.0
57.0 HLJ1970
62.2 HLJ384
67.6 CAFS227
81.3 HLJE298
83.7 HLJE27
108.2 [redacted]

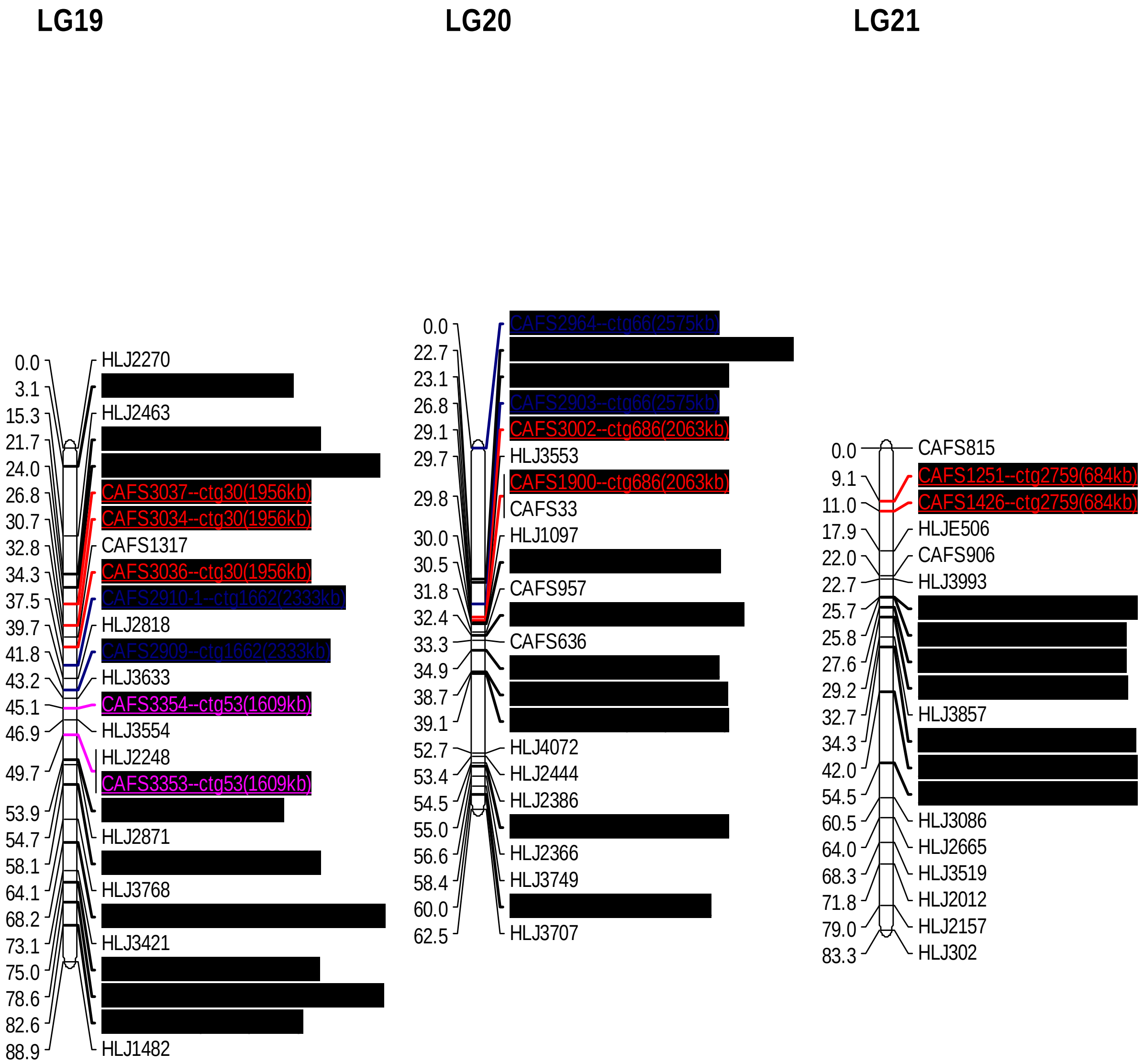

LG19
LG20
LG21
0.0
3.1
15.3
21.7
24.0
26.8
30.7
32.8
34.3
37.5
39.7
41.8
43.2
45.1
46.9
49.7
53.9
54.7
58.1
64.1
68.2
73.1
75.0
78.6
82.6
88.9
HLJ2270
[redacted]
HLJ2463
[redacted]
CAFS3037--ctg30(1956kb)
CAFS3034--ctg30(1956kb)
CAFS1317
CAFS3036--ctg30(1956kb)
CAFS2910-1--ctg1662(2333kb)
HLJ2818
CAFS2909--ctg1662(2333kb)
HLJ3633
CAFS3354--ctg53(1609kb)
HLJ3554
HLJ2248
CAFS3353--ctg53(1609kb)
[redacted]
HLJ2871
[redacted]
HLJ3768
[redacted]
HLJ3421
[redacted]
HLJ1482
0.0
22.7
23.1
26.8
29.1
29.7
29.8
30.0
30.5
31.8
32.4
33.3
34.9
38.7
39.1
52.7
53.4
54.5
55.0
56.6
58.4
60.0
62.5
CAFS2964--ctg66(2575kb)
[redacted]
CAFS2903--ctg66(2575kb)
CAFS3002--ctg686(2063kb)
HLJ3553
CAFS1900--ctg686(2063kb)
CAFS33
HLJ1097
[redacted]
CAFS957
[redacted]
CAFS636
[redacted]
HLJ4072
HLJ2444
HLJ2386
[redacted]
HLJ2366
HLJ3749
[redacted]
HLJ3707
0.0
9.1
11.0
17.9
22.0
22.7
25.7
25.8
27.6
29.2
32.7
34.3
42.0
54.5
60.5
64.0
68.3
71.8
79.0
83.3
CAFS815
CAFS1251--ctg2759(684kb)
CAFS1426--ctg2759(684kb)
HLJE506
CAFS906
HLJ3993
[redacted]
HLJ3857
[redacted]
HLJ3086
HLJ2665
HLJ3519
HLJ2012
HLJ2157
HLJ302

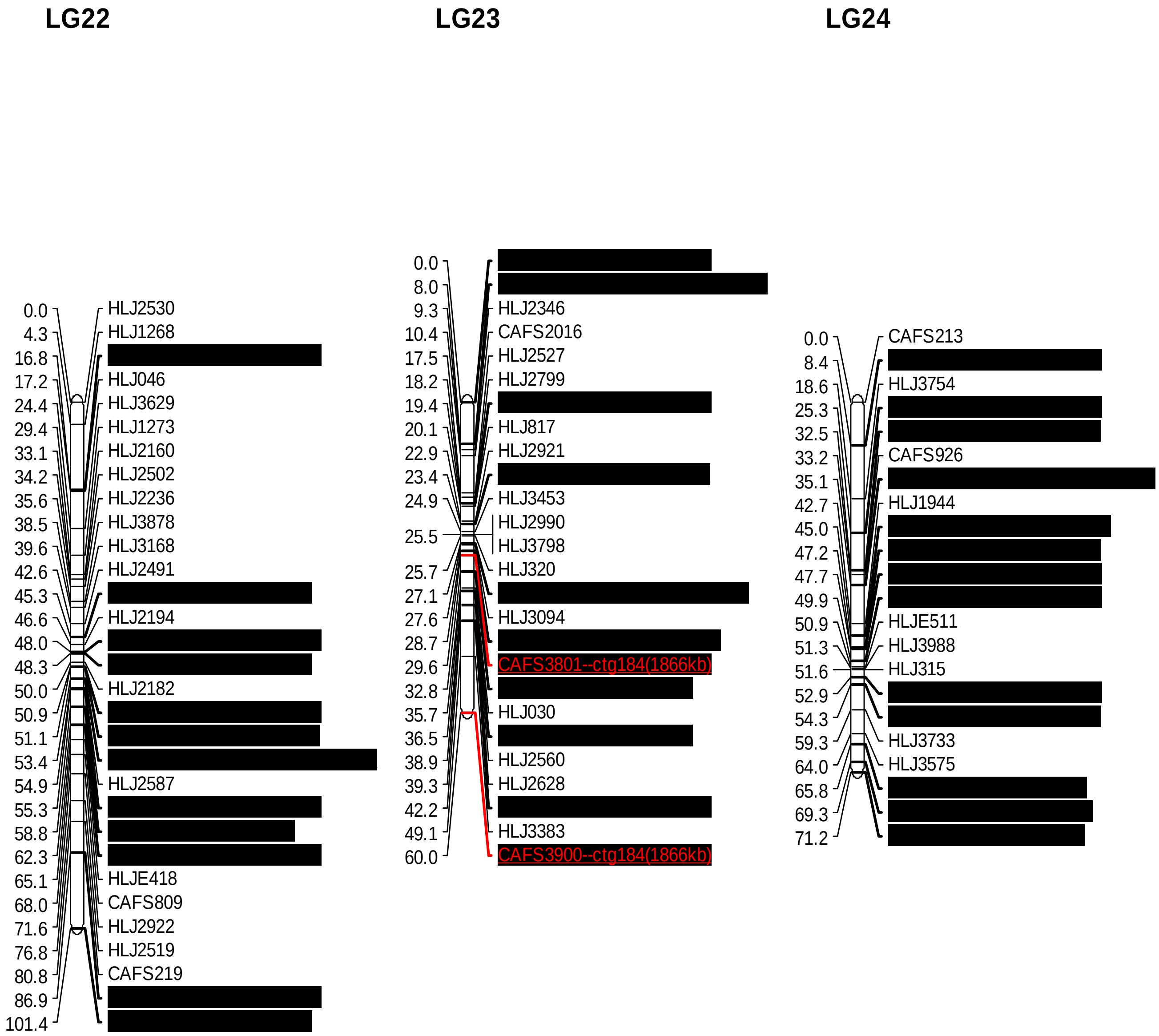
LG22
0.0 HLJ2530
4.3 HLJ1268
16.8 [redacted]
17.2 HLJ046
24.4 HLJ3629
29.4 HLJ1273
33.1 HLJ2160
34.2 HLJ2502
35.6 HLJ2236
38.5 HLJ3878
39.6 HLJ3168
42.6 HLJ2491
45.3 [redacted]
46.6 HLJ2194
48.0 [redacted]
48.3 [redacted]
50.0 HLJ2182
50.9 [redacted]
51.1 [redacted]
53.4 [redacted]
54.9 HLJ2587
55.3 [redacted]
58.8 [redacted]
62.3 [redacted]
65.1 HLJE418
68.0 CAFS809
71.6 HLJ2922
76.8 HLJ2519
80.8 CAFS219
86.9 [redacted]
101.4 [redacted]
LG23
0.0 [redacted]
8.0 [redacted]
9.3 HLJ2346
10.4 CAFS2016
17.5 HLJ2527
18.2 HLJ2799
19.4 [redacted]
20.1 HLJ817
22.9 HLJ2921
23.4 [redacted]
24.9 HLJ3453
25.5 HLJ2990
HLJ3798
25.7 HLJ320
27.1 [redacted]
27.6 HLJ3094
28.7 [redacted]
29.6 CAFS3801--ctg184(1866kb)
32.8 [redacted]
35.7 HLJ030
36.5 [redacted]
38.9 HLJ2560
39.3 HLJ2628
42.2 [redacted]
49.1 HLJ3383
60.0 CAFS3900--ctg184(1866kb)
LG24
0.0 CAFS213
8.4 [redacted]
18.6 HLJ3754
25.3 [redacted]
32.5 [redacted]
33.2 CAFS926
35.1 [redacted]
42.7 HLJ1944
45.0 [redacted]
47.2 [redacted]
47.7 [redacted]
49.9 [redacted]
50.9 HLJE511
51.3 HLJ3988
51.6 HLJ315
52.9 [redacted]
54.3 [redacted]
59.3 HLJ3733
64.0 HLJ3575
65.8 [redacted]
69.3 [redacted]
71.2 [redacted]

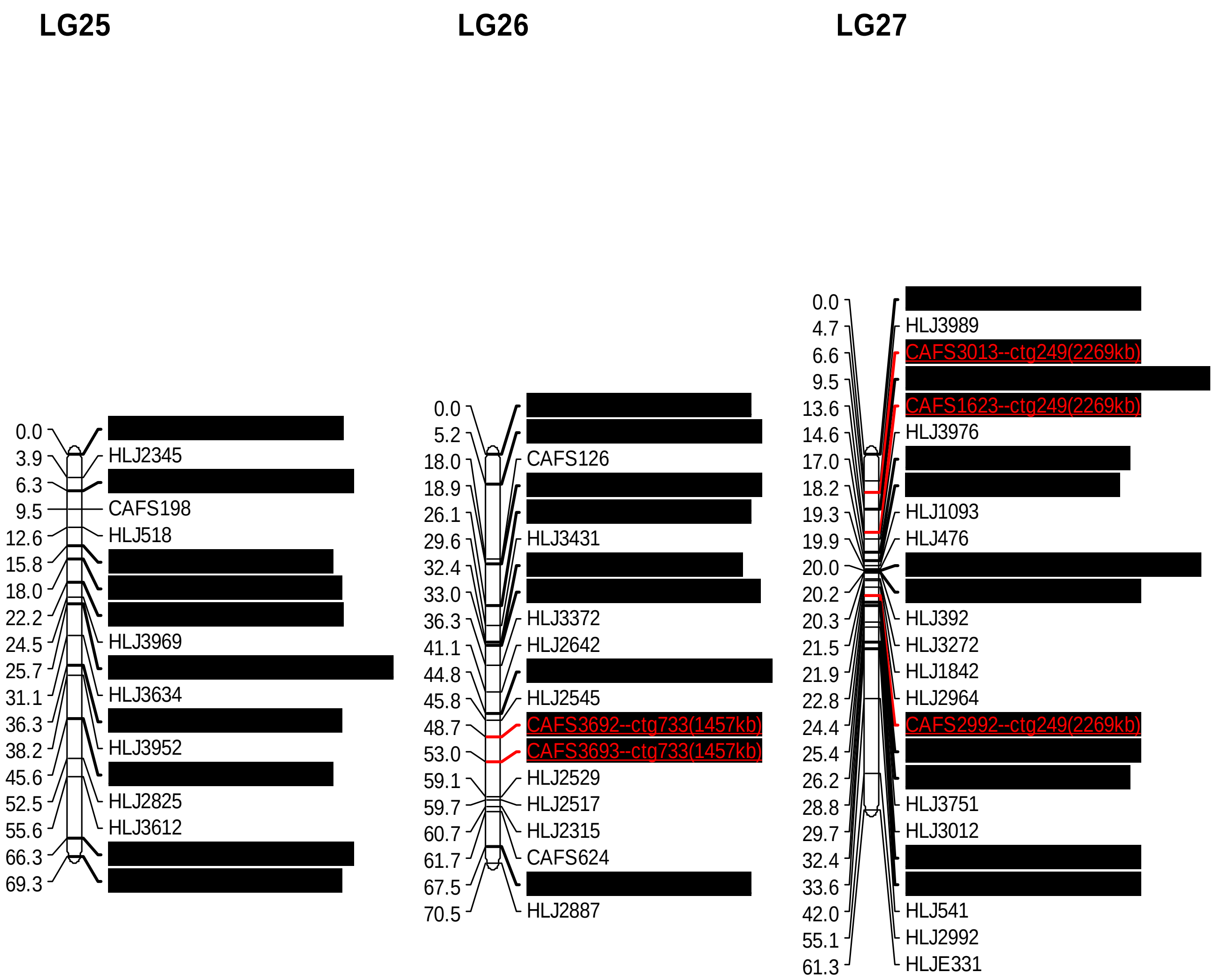
LG25
0.0 [redacted]
3.9 HLJ2345
6.3 [redacted]
9.5 CAFS198
12.6 HLJ518
15.8 [redacted]
18.0 [redacted]
22.2 [redacted]
24.5 HLJ3969
25.7 [redacted]
31.1 HLJ3634
36.3 [redacted]
38.2 HLJ3952
45.6 [redacted]
52.5 HLJ2825
55.6 HLJ3612
66.3 [redacted]
69.3 [redacted]
LG26
0.0 [redacted]
5.2 [redacted]
18.0 CAFS126
18.9 [redacted]
26.1 [redacted]
29.6 HLJ3431
32.4 [redacted]
33.0 [redacted]
36.3 HLJ3372
41.1 HLJ2642
44.8 [redacted]
45.8 HLJ2545
48.7 CAFS3692--ctg733(1457kb)
53.0 CAFS3693--ctg733(1457kb)
59.1 HLJ2529
59.7 HLJ2517
60.7 HLJ2315
61.7 CAFS624
67.5 [redacted]
70.5 HLJ2887
LG27
0.0 [redacted]
4.7 HLJ3989
6.6 CAFS3013--ctg249(2269kb)
9.5 [redacted]
13.6 CAFS1623--ctg249(2269kb)
14.6 HLJ3976
17.0 [redacted]
18.2 [redacted]
19.3 HLJ1093
19.9 HLJ476
20.0 [redacted]
20.2 [redacted]
20.3 HLJ392
21.5 HLJ3272
21.9 HLJ1842
22.8 HLJ2964
24.4 CAFS2992--ctg249(2269kb)
25.4 [redacted]
26.2 [redacted]
28.8 HLJ3751
29.7 HLJ3012
32.4 [redacted]
33.6 [redacted]
42.0 HLJ541
55.1 HLJ2992
61.3 HLJE331

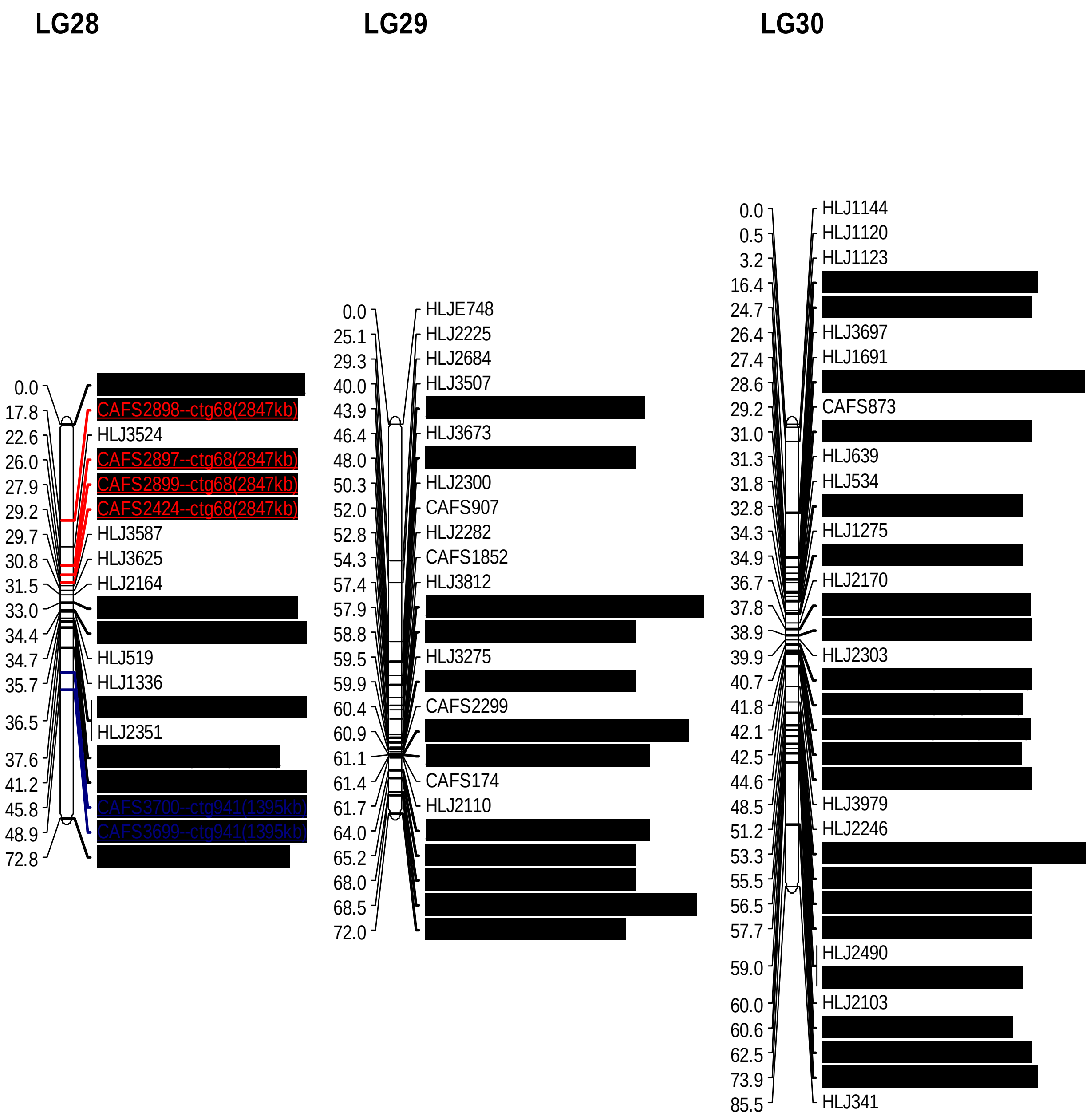

LG28
0.0
17.8
22.6
26.0
27.9
29.2
29.7
30.8
31.5
33.0
34.4
34.7
35.7
36.5
37.6
41.2
45.8
48.9
72.8
[redacted]
CAFS2898--ctg68(2847kb)
HLJ3524
CAFS2897--ctg68(2847kb)
CAFS2899--ctg68(2847kb)
CAFS2424--ctg68(2847kb)
HLJ3587
HLJ3625
HLJ2164
[redacted]
HLJ519
HLJ1336
[redacted]
HLJ2351
[redacted]
CAFS3700--ctg941(1395kb)
CAFS3699--ctg941(1395kb)
[redacted]
LG29
0.0
25.1
29.3
40.0
43.9
46.4
48.0
50.3
52.0
52.8
54.3
57.4
57.9
58.8
59.5
59.9
60.4
60.9
61.1
61.4
61.7
64.0
65.2
68.0
68.5
72.0
HLJE748
HLJ2225
HLJ2684
HLJ3507
[redacted]
HLJ3673
[redacted]
HLJ2300
CAFS907
HLJ2282
CAFS1852
HLJ3812
[redacted]
HLJ3275
[redacted]
CAFS2299
[redacted]
CAFS174
HLJ2110
[redacted]
LG30
0.0
0.5
3.2
16.4
24.7
26.4
27.4
28.6
29.2
31.0
31.3
31.8
32.8
34.3
34.9
36.7
37.8
38.9
39.9
40.7
41.8
42.1
42.5
44.6
48.5
51.2
53.3
55.5
56.5
57.7
59.0
60.0
60.6
62.5
73.9
85.5
HLJ1144
HLJ1120
HLJ1123
[redacted]
HLJ3697
HLJ1691
[redacted]
CAFS873
[redacted]
HLJ639
HLJ534
[redacted]
HLJ1275
[redacted]
HLJ2170
[redacted]
HLJ2303
[redacted]
HLJ3979
HLJ2246
[redacted]
HLJ2490
[redacted]
HLJ2103
[redacted]
HLJ341

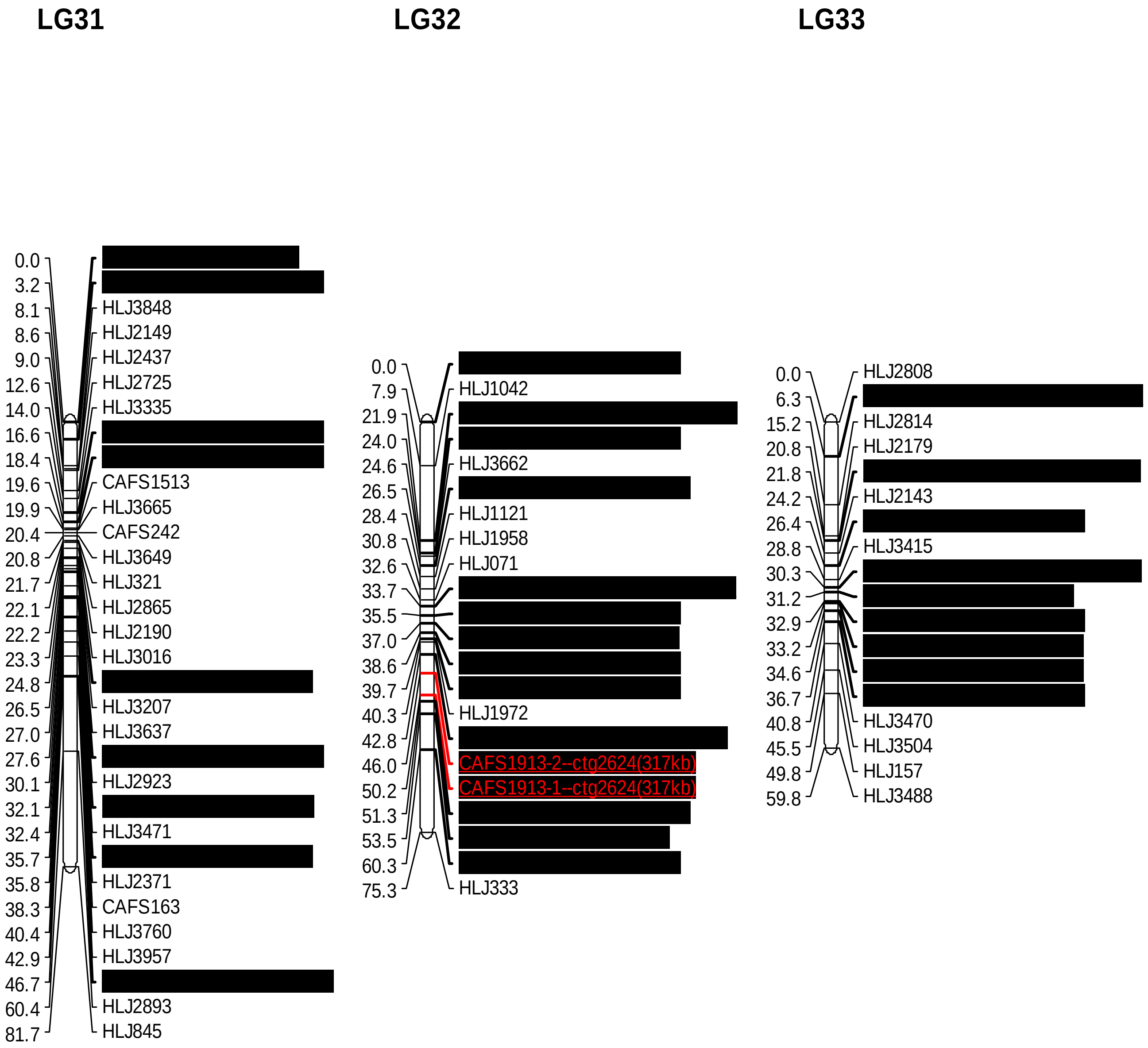
LG31
0.0
3.2
8.1
8.6
9.0
12.6
14.0
16.6
18.4
19.6
19.9
20.4
20.8
21.7
22.1
22.2
23.3
24.8
26.5
27.0
27.6
30.1
32.1
32.4
35.7
35.8
38.3
40.4
42.9
46.7
60.4
81.7
[redacted]
HLJ3848
HLJ2149
HLJ2437
HLJ2725
HLJ3335
[redacted]
CAFS1513
HLJ3665
CAFS242
HLJ3649
HLJ321
HLJ2865
HLJ2190
HLJ3016
[redacted]
HLJ3207
HLJ3637
[redacted]
HLJ2923
[redacted]
HLJ3471
[redacted]
HLJ2371
CAFS163
HLJ3760
HLJ3957
[redacted]
HLJ2893
HLJ845
LG32
0.0
7.9
21.9
24.0
24.6
26.5
28.4
30.8
32.6
33.7
35.5
37.0
38.6
39.7
40.3
42.8
46.0
50.2
51.3
53.5
60.3
75.3
[redacted]
HLJ1042
[redacted]
HLJ3662
[redacted]
HLJ1121
HLJ1958
HLJ071
[redacted]
HLJ1972
[redacted]
CAFS1913-2--ctg2624(317kb)
CAFS1913-1--ctg2624(317kb)
[redacted]
HLJ333
LG33
0.0
6.3
15.2
20.8
21.8
24.2
26.4
28.8
30.3
31.2
32.9
33.2
34.6
36.7
40.8
45.5
49.8
59.8
HLJ2808
[redacted]
HLJ2814
HLJ2179
[redacted]
HLJ2143
[redacted]
HLJ3415
[redacted]
HLJ3470
HLJ3504
HLJ157
HLJ3488

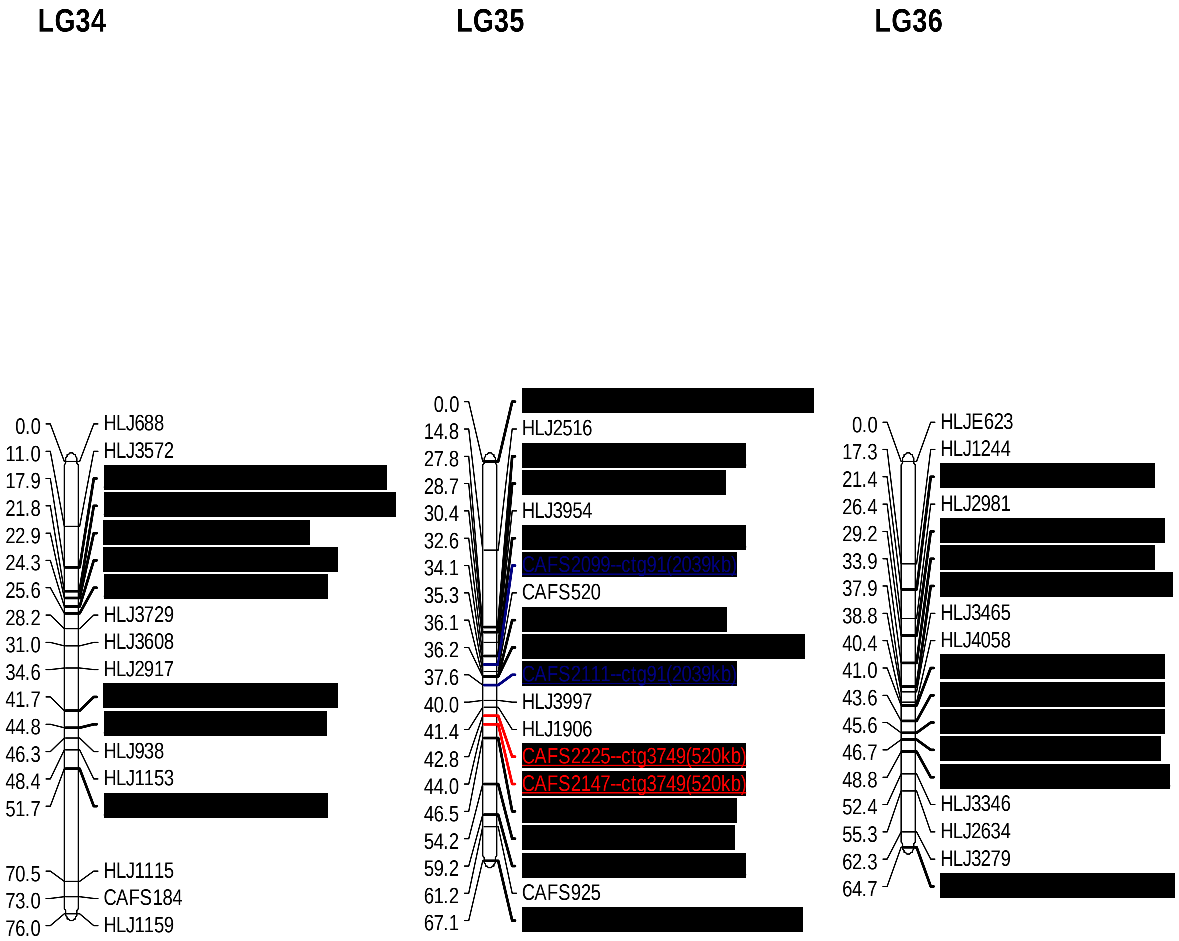
LG34
0.0 HLJ688
11.0 HLJ3572
17.9 [redacted]
21.8 [redacted]
22.9 [redacted]
24.3 [redacted]
25.6 [redacted]
28.2 HLJ3729
31.0 HLJ3608
34.6 HLJ2917
41.7 [redacted]
44.8 [redacted]
46.3 HLJ938
48.4 HLJ1153
51.7 [redacted]
70.5 HLJ1115
73.0 CAFS184
76.0 HLJ1159
LG35
0.0 [redacted]
14.8 HLJ2516
27.8 [redacted]
28.7 [redacted]
30.4 HLJ3954
32.6 [redacted]
34.1 [redacted]
35.3 CAFS520
36.1 [redacted]
36.2 [redacted]
37.6 [redacted]
40.0 HLJ3997
41.4 HLJ1906
42.8 [redacted]
44.0 [redacted]
46.5 [redacted]
54.2 [redacted]
59.2 [redacted]
61.2 CAFS925
67.1 [redacted]
LG36
0.0 HLJE623
17.3 HLJ1244
21.4 [redacted]
26.4 HLJ2981
29.2 [redacted]
33.9 [redacted]
37.9 [redacted]
38.8 HLJ3465
40.4 HLJ4058
41.0 [redacted]
43.6 [redacted]
45.6 [redacted]
46.7 [redacted]
48.8 [redacted]
52.4 HLJ3346
55.3 HLJ2634
62.3 HLJ3279
64.7 [redacted]

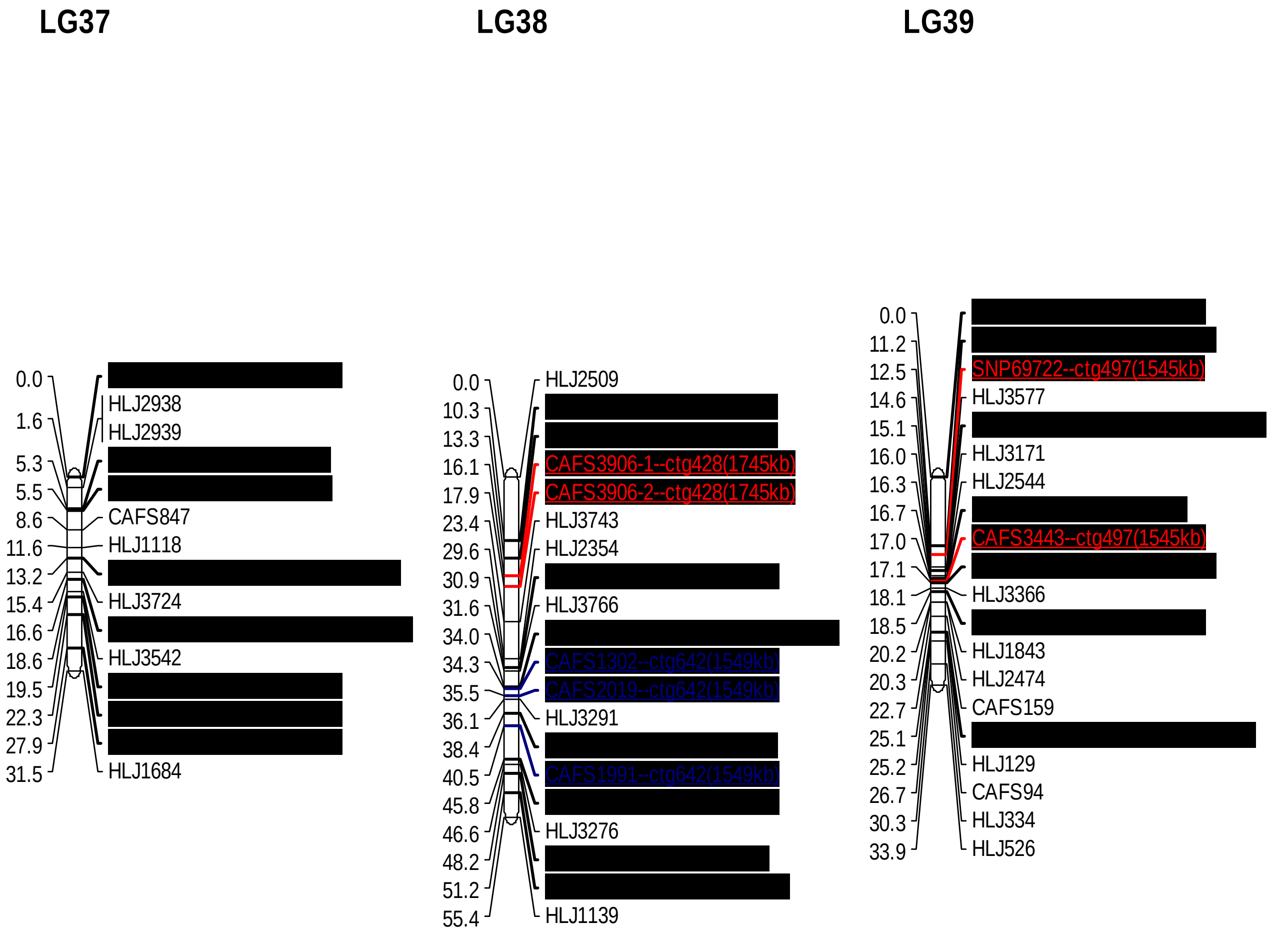
LG37
0.0
1.6
5.3
5.5
8.6
11.6
13.2
15.4
16.6
18.6
19.5
22.3
27.9
31.5
[redacted]
HLJ2938
HLJ2939
[redacted]
CAFS847
HLJ1118
[redacted]
HLJ3724
[redacted]
HLJ3542
[redacted]
HLJ1684
LG38
0.0
10.3
13.3
16.1
17.9
23.4
29.6
30.9
31.6
34.0
34.3
35.5
36.1
38.4
40.5
45.8
46.6
48.2
51.2
55.4
HLJ2509
[redacted]
CAFS3906-1--ctg428(1745kb)
CAFS3906-2--ctg428(1745kb)
HLJ3743
HLJ2354
[redacted]
HLJ3766
[redacted]
CAFS1302--ctg642(1549kb)
CAFS2019--ctg642(1549kb)
HLJ3291
[redacted]
CAFS1991--ctg642(1549kb)
[redacted]
HLJ3276
[redacted]
HLJ1139
LG39
0.0
11.2
12.5
14.6
15.1
16.0
16.3
16.7
17.0
17.1
18.1
18.5
20.2
20.3
22.7
25.1
25.2
26.7
30.3
33.9
[redacted]
SNP69722--ctg497(1545kb)
HLJ3577
[redacted]
HLJ3171
HLJ2544
[redacted]
CAFS3443--ctg497(1545kb)
[redacted]
HLJ3366
[redacted]
HLJ1843
HLJ2474
CAFS159
[redacted]
HLJ129
CAFS94
HLJ334
HLJ526

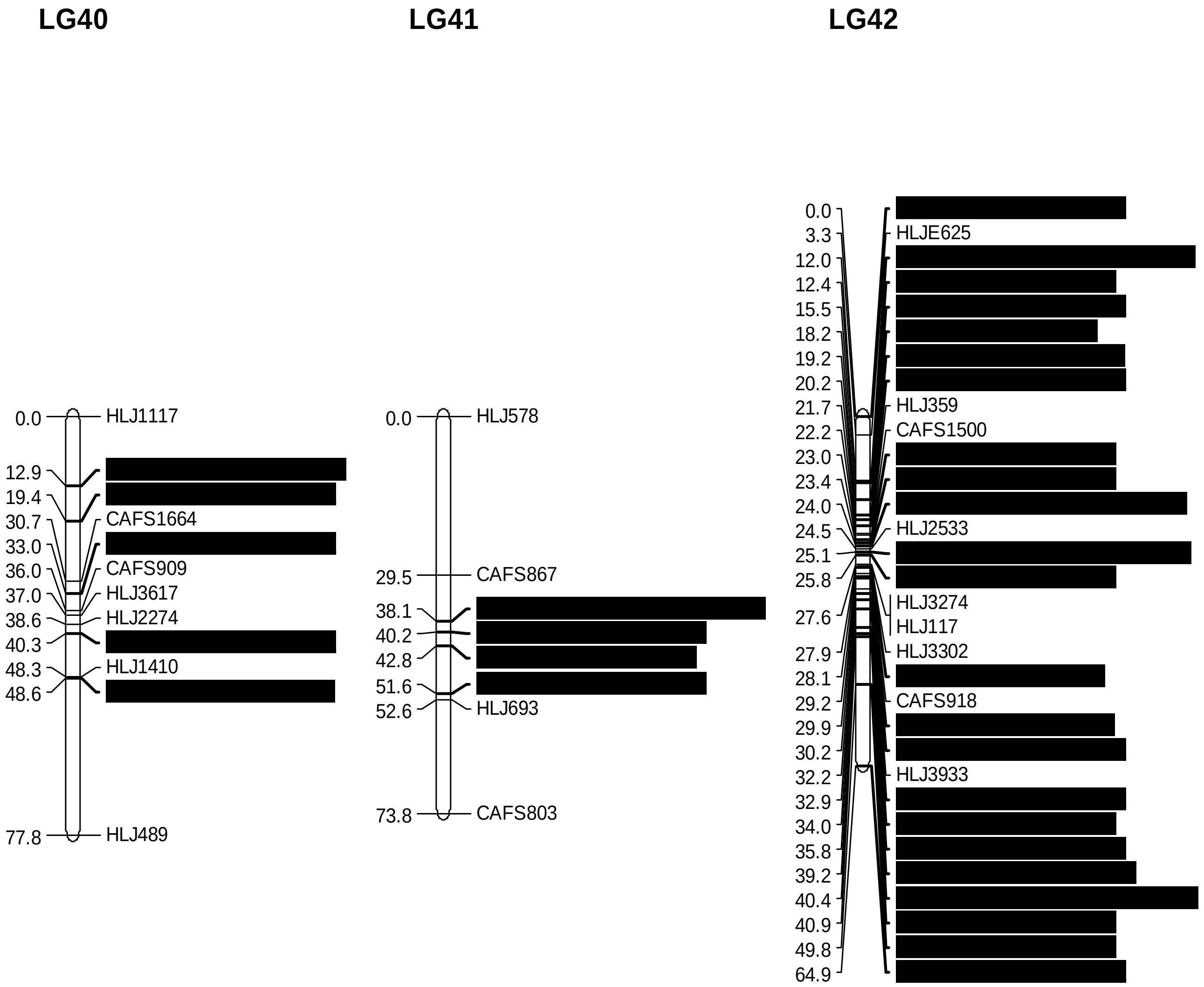
LG40
0.0 HLJ1117
12.9 [redacted]
19.4 [redacted]
30.7 CAFS1664
33.0 [redacted]
36.0 CAFS909
37.0 HLJ3617
38.6 HLJ2274
40.3 [redacted]
48.3 HLJ1410
48.6 [redacted]
77.8 HLJ489
LG41
0.0 HLJ578
29.5 CAFS867
38.1 [redacted]
40.2 [redacted]
42.8 [redacted]
51.6 [redacted]
52.6 HLJ693
73.8 CAFS803
LG42
0.0 [redacted]
3.3 HLJE625
12.0 [redacted]
12.4 [redacted]
15.5 [redacted]
18.2 [redacted]
19.2 [redacted]
20.2 [redacted]
21.7 HLJ359
22.2 CAFS1500
23.0 [redacted]
23.4 [redacted]
24.0 [redacted]
24.5 HLJ2533
25.1 [redacted]
25.8 [redacted]
27.6 HLJ3274
HLJ117
27.9 HLJ3302
28.1 [redacted]
29.2 CAFS918
29.9 [redacted]
30.2 [redacted]
32.2 HLJ3933
32.9 [redacted]
34.0 [redacted]
35.8 [redacted]
39.2 [redacted]
40.4 [redacted]
40.9 [redacted]
49.8 [redacted]
64.9 [redacted]

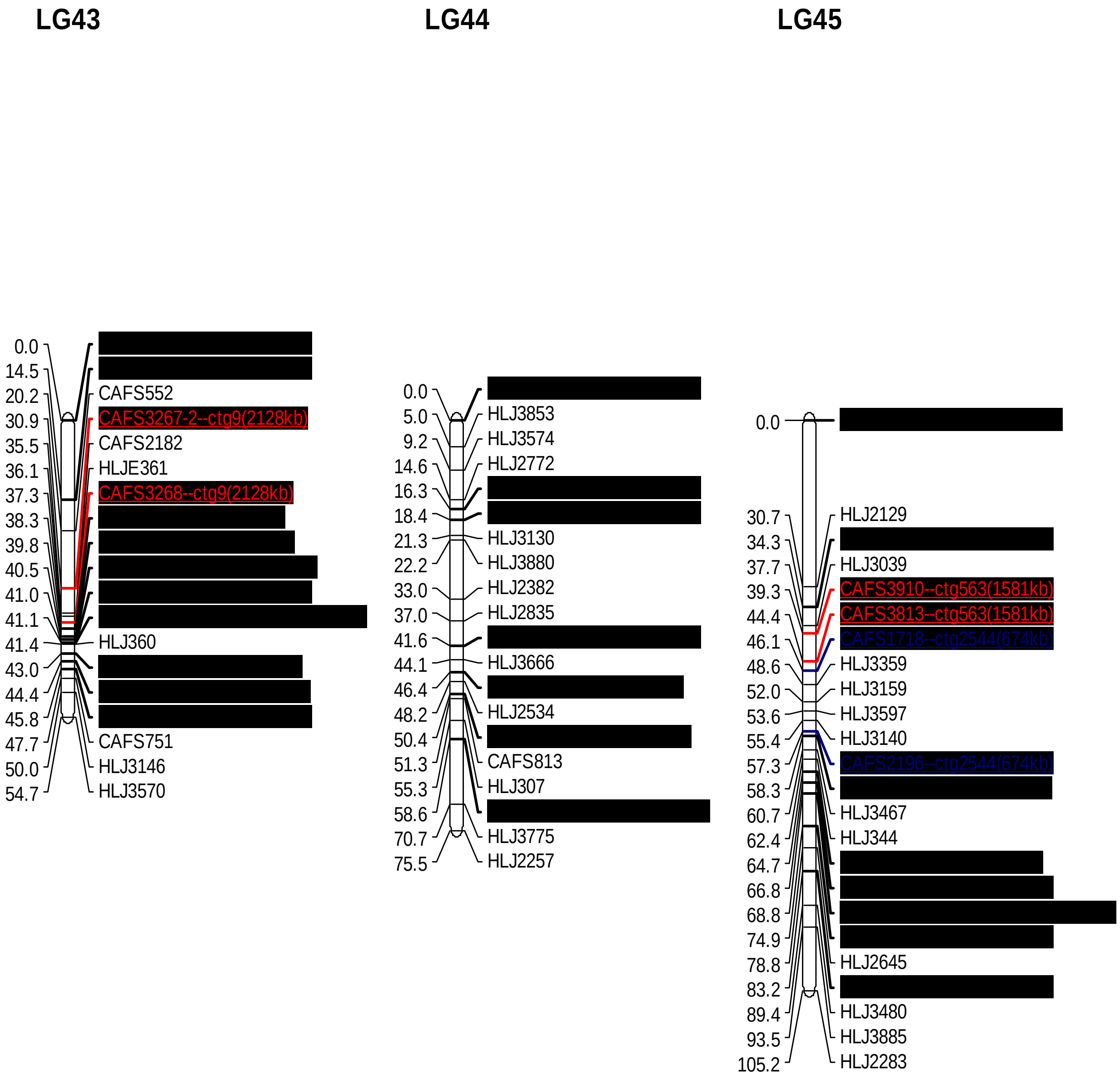
LG43
0.0 [redacted]
14.5 [redacted]
20.2 CAFS552
30.9 CAFS3267-2--ctg9(2128kb)
35.5 CAFS2182
36.1 HLJE361
37.3 CAFS3268--ctg9(2128kb)
38.3 [redacted]
39.8 [redacted]
40.5 [redacted]
41.0 [redacted]
41.1 [redacted]
41.4 HLJ360
43.0 [redacted]
44.4 [redacted]
45.8 [redacted]
47.7 CAFS751
50.0 HLJ3146
54.7 HLJ3570
LG44
0.0 [redacted]
5.0 HLJ3853
9.2 HLJ3574
14.6 HLJ2772
16.3 [redacted]
18.4 [redacted]
21.3 HLJ3130
22.2 HLJ3880
33.0 HLJ2382
37.0 HLJ2835
41.6 [redacted]
44.1 HLJ3666
46.4 [redacted]
48.2 HLJ2534
50.4 [redacted]
51.3 CAFS813
55.3 HLJ307
58.6 [redacted]
70.7 HLJ3775
75.5 HLJ2257
LG45
0.0 [redacted]
30.7 HLJ2129
34.3 [redacted]
37.7 HLJ3039
39.3 CAFS3910--ctg563(1581kb)
44.4 CAFS3813--ctg563(1581kb)
46.1 CAFS1718--ctg2544(674kb)
48.6 HLJ3359
52.0 HLJ3159
53.6 HLJ3597
55.4 HLJ3140
57.3 CAFS2196--ctg2544(674kb)
58.3 [redacted]
60.7 HLJ3467
62.4 HLJ344
64.7 [redacted]
66.8 [redacted]
68.8 [redacted]
74.9 [redacted]
78.8 HLJ2645
83.2 [redacted]
89.4 HLJ3480
93.5 HLJ3885
105.2 HLJ2283

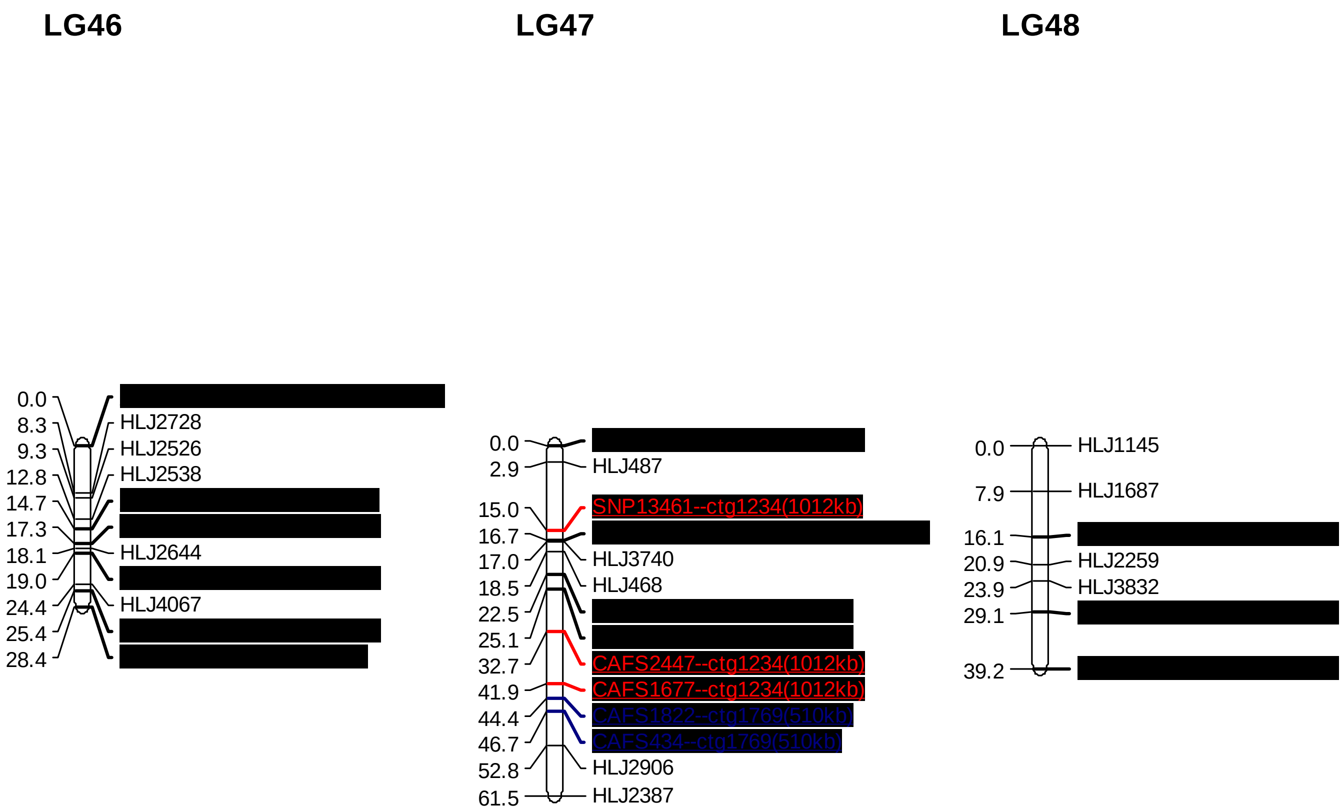
LG46
LG47
LG48
0.0 [redacted]
8.3 HLJ2728
9.3 HLJ2526
12.8 HLJ2538
14.7 [redacted]
17.3 [redacted]
18.1 HLJ2644
19.0 [redacted]
24.4 HLJ4067
25.4 [redacted]
28.4 [redacted]
0.0 [redacted]
2.9 HLJ487
15.0 SNP13461--ctg1234(1012kb)
16.7 [redacted]
17.0 HLJ3740
18.5 HLJ468
22.5 [redacted]
25.1 [redacted]
32.7 CAFS2447--ctg1234(1012kb)
41.9 CAFS1677--ctg1234(1012kb)
44.4 CAFS1822--ctg1769(510kb)
46.7 CAFS434--ctg1769(510kb)
52.8 HLJ2906
61.5 HLJ2387
0.0 HLJ1145
7.9 HLJ1687
16.1 [redacted]
20.9 HLJ2259
23.9 HLJ3832
29.1 [redacted]
39.2 [redacted]

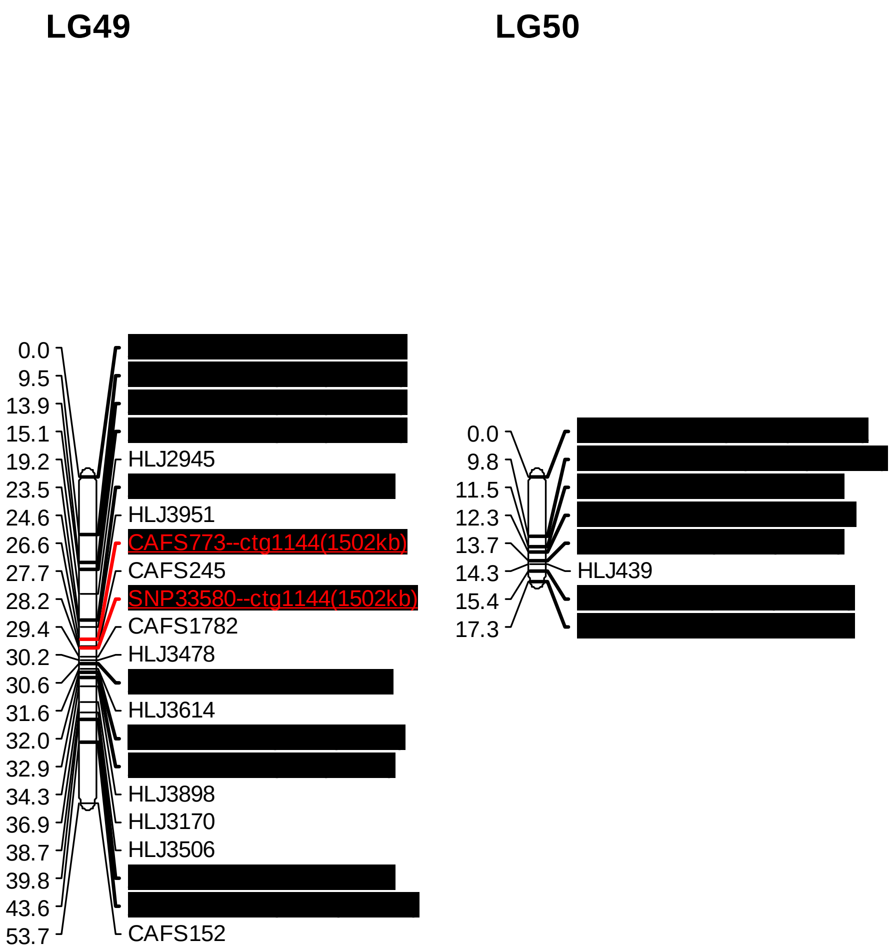
LG49
LG50
0.0 [redacted]
9.5 [redacted]
13.9 [redacted]
15.1 [redacted]
19.2 HLJ2945
23.5 [redacted]
24.6 HLJ3951
26.6 CAFS773--ctg1144(1502kb)
27.7 CAFS245
28.2 SNP33580--ctg1144(1502kb)
29.4 CAFS1782
30.2 HLJ3478
30.6 [redacted]
31.6 HLJ3614
32.0 [redacted]
32.9 [redacted]
34.3 HLJ3898
36.9 HLJ3170
38.7 HLJ3506
39.8 [redacted]
43.6 [redacted]
53.7 CAFS152
0.0 [redacted]
9.8 [redacted]
11.5 [redacted]
12.3 [redacted]
13.7 [redacted]
14.3 HLJ439
15.4 [redacted]
17.3 [redacted]
